# Dust, Sand, and Winds Within an Active Martian Storm in Jezero Crater

**DOI:** 10.1029/2022GL100126

**Published:** 2022-09-09

**Authors:** M. T. Lemmon, M. D. Smith, D. Viudez‐Moreiras, M. de la Torre‐Juarez, A. Vicente‐Retortillo, A. Munguira, A. Sanchez‐Lavega, R. Hueso, G. Martinez, B. Chide, R. Sullivan, D. Toledo, L. Tamppari, T. Bertrand, J. F. Bell, C. Newman, M. Baker, D. Banfield, J. A. Rodriguez‐Manfredi, J. N. Maki, V. Apestigue

**Affiliations:** ^1^ Space Science Institute Boulder CO USA; ^2^ NASA Goddard Space Flight Center Greenbelt MD USA; ^3^ Centro de Astrobiologia (INTA‐CSIC) Madrid Spain; ^4^ Jet Propulsion Laboratory California Institute of Technology Pasadena CA USA; ^5^ Física Aplicada, Escuela de Ingeniería de Bilbao UPV/EHU Bilbao Spain; ^6^ Lunar and Planetary Institute Houston TX USA; ^7^ Space and Planetary Exploration Team Los Alamos National Laboratory Los Alamos NM USA; ^8^ Cornell University Ithaca NY USA; ^9^ Instituto Nacional de Técnica Aerospacial Madrid Spain; ^10^ LESIA, Observatoire de Paris Meudon France; ^11^ Arizona State University Tempe AZ USA; ^12^ Aeolis Research Chandler AZ USA; ^13^ Smithsonian National Air and Space Museum Washington DC USA

## Abstract

Rovers and landers on Mars have experienced local, regional, and planetary‐scale dust storms. However, in situ documentation of active lifting within storms has remained elusive. Over 5–11 January 2022 (L_S_ 153°–156°), a dust storm passed over the Perseverance rover site. Peak visible optical depth was ∼2, and visibility across the crater was briefly reduced. Pressure amplitudes and temperatures responded to the storm. Winds up to 20 m s^−1^ rotated around the site before the wind sensor was damaged. The rover imaged 21 dust‐lifting events—gusts and dust devils—in one 25‐min period, and at least three events mobilized sediment near the rover. Rover tracks and drill cuttings were extensively modified, and debris was moved onto the rover deck. Migration of small ripples was seen, but there was no large‐scale change in undisturbed areas. This work presents an overview of observations and initial results from the study of the storm.

## Key Points

- The Perseverance rover documented the meteorology and effects of a dust storm as it passed over Jezero crater, Mars
- The storm brought damaging winds and wide‐spread dust lifting, while modifying the pressure amplitudes and thermal cycle at the site
- Winds extensively modified previously disturbed areas, while sand motion and small‐scale ripple migration occurred all around the rover

## 1 Introduction

Martian dust storms are a key part of the current climate that remain poorly predictable (Newman & Richardson, 2015). They range in scale from local, to regional, to planet‐encircling dust events (PEDE). Orbital sounding and imagery have been used to track and classify storms, showing patterns in where and when storms occur and move (Cantor et al., 2001; Wang & Richardson, 2015). Development of storms has been associated to the activity of Kelvin waves (Tillman, 1988), baroclinic waves (Battalio et al., 2016; James et al., 1999; Sánchez‐Lavega et al., 2022b), resonant interaction with pressure tides (Leovy & Zurek, 1979; Wang et al., 2003), or combinations of these mechanisms depending on the time of the season (Zurita‐Zurita et al., 2022). Yet there is no predictor of why one local storm grows to regional scale and another dies, or one regional storm triggers or joins a PEDE and another dissipates.

*In situ* data on storm processes is necessary for developing a complete picture of storm evolution. While landers and rovers have experienced dust storms, there has been no prior comprehensive collection of meteorological data within the active lifting area of a storm. The *Viking* landers (VL) documented the effects of planetary scale events, including pressure and winds associated with storm onset, without documenting local lifting or activity (Hess et al., 1980; Ryan & Henry, 1979). The Spirit and Opportunity rovers, without meteorological capabilities, experienced local, regional, and planetary events that were associated with a reduction in local dust lifting (Greeley et al., 2010; M. Lemmon et al., 2015). The *Curiosity* rover experienced optical depths >8 yet saw a reduction in local wind and dust lifting activity and did not see an unusual amount of sediment motion (Guzewich et al., 2019; Ordoñez‐Etxeberria et al., 2020; Viúdez‐Moreiras et al., 2019). *InSight* obtained meteorological documentation of a large dust storm but did not record local dust lifting activity (Viúdez‐Moreiras et al., 2020).

Over 5–11 January 2022 (L_S_ 153°–156°), a large regional dust storm affected the *Perseverance*, *InSight*, and *Curiosity* sites (Malin & Cantor, 2022b). We report on the events at the *Perseverance* site in Jezero crater: six sols (Martian solar days of 24h40’ duration) of dynamic and dusty weather that erased rover tracks, cleaned some surfaces, dirtied others, and partly damaged the rover's wind sensors.

## 2 Data and Methods

The *Perseverance* rover landed on 19 February 2021 in Jezero crater (sol 0, L_S_ 6°), at 77.5°E longitude, 18.4°N latitude. Among its instruments were several cameras and a meteorology package. Mastcam‐Z is a multispectral, stereo camera pair with zoom lenses (J. F. Bell et al., 2021). Navcam is a color, stereo camera pair with a wide field of view (FOV) and the front and rear Hazcams are each color, stereo camera pairs (J. N. Maki et al., 2020). MEDA, the Mars Environmental Dynamics Analyzer, is a meteorological package that measures pressure, temperature, winds, ultraviolet to infrared radiation, and humidity (Rodriguez‐Manfredi et al., 2021). Its Radiation and Dust Sensor (RDS) includes a zenith‐looking camera, Skycam (Apestigue et al., 2022). SuperCam imaged targets at the site with the Remote Microscopic Imager (RMI) as well as recording sounds at the site with its microphone (Maurice et al., 2021, 2022).

### 2.1 Meteorology

MEDA functioned independently of rover wake‐sleep cycles at a cadence specified in observation tables that were typically updated daily. Typical behavior was to measure with all sensors (excluding the camera) at 1 Hz, at least 5 min of each hour and to run continuously at 1 Hz during even hours on even sols and odd hours on odd sols. Running duration was limited by power availability; availability of adequate downlink data volume; a need to stop acquisition to transfer data to the rover; and, for only the wind sensor, a need to refrain from collecting data while the rover communicated data to Earth via satellite. Additional data collection was commanded during the storm.

### 2.2 Optical Depth

Mastcam‐Z measured column optical depth via Sun imaging using solar filters (neutral density filters combined with a narrow 880‐nm filter for the right camera and a short‐pass filter to get red, green, and blue optical depths for the left camera). Solar images were calibrated to radiance (Hayes et al., 2021) and solar flux was calculated and used to determine atmospheric extinction (see also M. Lemmon et al., 2015).

Skycam's 126° diameter view of the sky is partly obscured by a neutral density annulus, which allowed direct solar imaging in mid‐morning and mid‐afternoon. Due to the limited range of solar elevations, Skycam optical depths were calibrated to match Mastcam‐Z near‐infrared optical depths using measurements close in time.

MEDA sensor data were also used to determine optical depth. Thermal Infrared Radiometer (TIRS) measures broadband and 15‐μm radiance (Sebastián et al., 2021), which have been used to derive 9‐μm dust optical depth (Smith et al., 2022). The RDS's Top‐7 photodiode measures broad‐band solar downward flux that was calibrated to optical depth using a simple 2‐stream model to tune the results to match the Mastcam‐Z 880‐nm channel. Dust optical depth varies by only a few percent across the visible and very‐near infrared (M. T. Lemmon et al., 2019).

### 2.3 Site Imaging

Upon arrival at the *Issole* sample‐collection site on sol 286, the rover acquired a site panorama including the rover deck with Navcam, as well as images of the area in front of and behind the rover with Hazcams. During the next sols, a site panorama was acquired with Mastcam‐Z. All the scene was acquired with the 34‐mm zoom setting; many areas were imaged at zooms as high as 110‐mm. Hazcam images were acquired episodically to support contact and proximity science operations. Once the storm was recognized, additional Hazcams were acquired to assess the storm's impact. In addition, the Navcam and the 34‐mm Mastcam‐Z site panoramas were repeated after the storm.

Navcam acquired quasiperiodic environmental survey images. These included dust devil surveys (DDS), which used 3 monochrome images at each of 5 aims to survey the complete horizon for motion; dust devil movies (DDM) which used 21–45 monochrome exposures at a single aim to find and track motion; and cloud surveys, which used 5 color images to mosaic nearly the full sky hemisphere.

## 3 Results

### 3.1 Progression of the Storm

**Figure 1:**
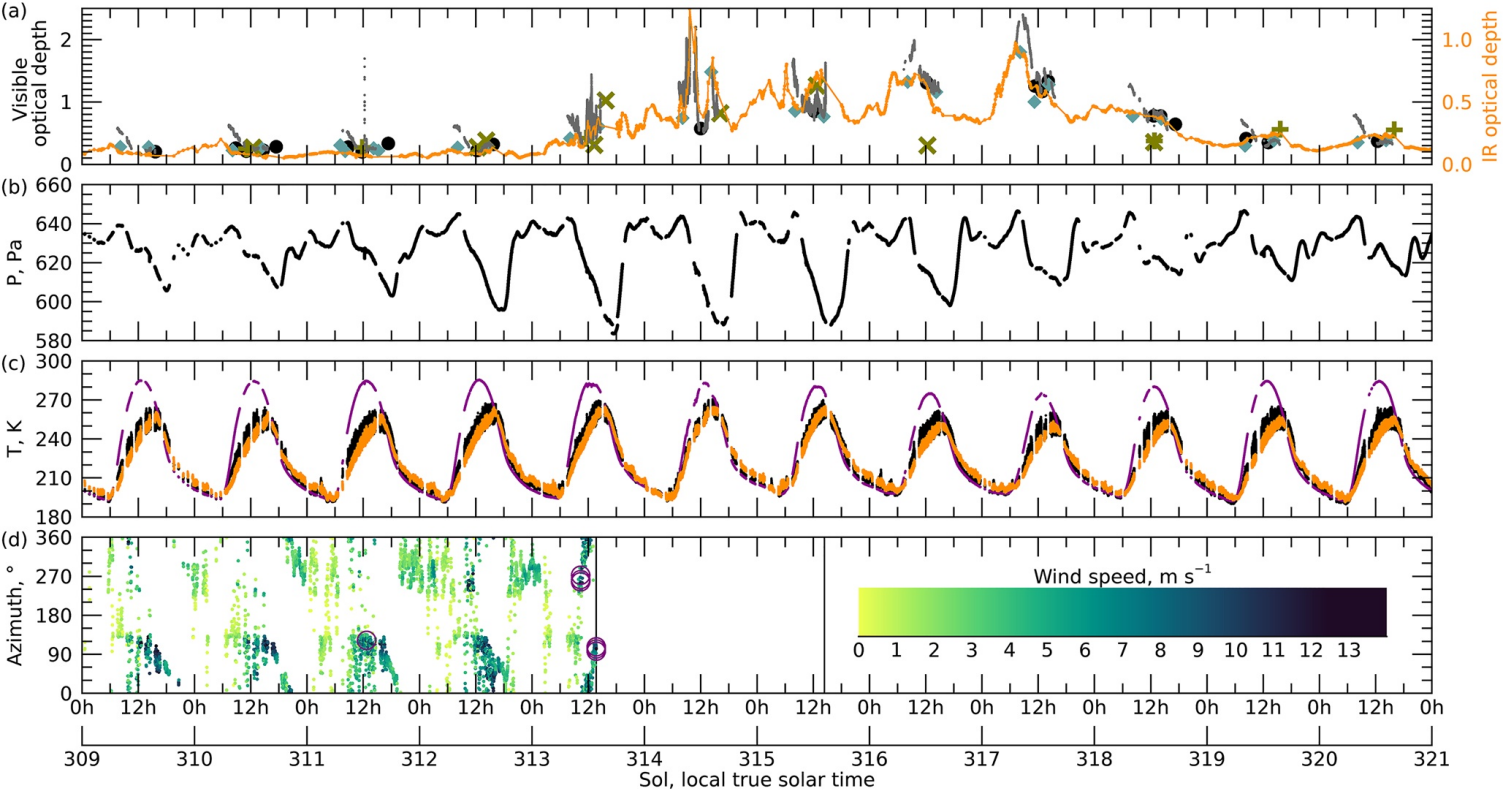
Meteorology summary from the start of sol 309 to the end of sol 321: (a) Visible optical depth from Mastcam‐Z (black circles), Skycam (blue diamonds), Radiation and Dust Sensor (slate gray line fragments); Navcam line‐of‐sight extinction per 10‐km (olive x and + for color and gray‐scale images); and 9‐μm optical depth from Thermal Infrared Radiometer (orange, scale 2x different); (b) pressure; (c) temperature for surface (magenta), 1.45‐m air (black), and lowest 200 m of the atmosphere (orange); (d) wind direction, with 1‐min average speed shown via color‐bar from 0 to 13 m s^−1^, circles indicating gusts >15 m s^−1^, and vertical lines showing the two boom failures. Relative uncertainties are typically smaller than the size of the symbols.

From 3 January 2022 (sol 310, L_S_ 151.8°), a large regional storm moved across the equator southwest of the rover and expanded eastward (Malin & Cantor, 2022b). Unusual dust‐lifting activity was noticed around the rover on sol 313 (L_S_ 153.4°), and optical depths increased then, through sol 318 (L_S_ 156.0°). After skies cleared at the Perseverance site, the storm gradually abated to the east over the next sols (Malin & Cantor, 2022a). (see Figure S1 in Supporting Information S1) Extra environmental monitoring was included in sol 313 and subsequent plans, but the rover also continued with rock‐coring operations. Figure 1 shows key meteorological measurements through the storm.

### 3.2 Optical Depth

Prior to the storm, visible optical depth was near 0.3 and starting to increase from the seasonal minimum (Figure 1a). Mornings had higher optical depths by 0.1–0.2 partly due to ice hazes and clouds (M. T. Lemmon et al., 2022). 9‐μm dust optical depth was approximately half the visible optical depth and varied little (Smith et al., 2022). The apparent high optical depth seen by RDS for a dust devil near noon on sol 311 was an artifact but serves as a marker for the event.

Elevated and variable optical depths were seen beginning sol 313. Extinction optical depths from solar images was only slightly elevated for two samples on sol 313; but RDS and TIRS showed rapid excursions with factor‐of‐several changes and peak optical depths of 1.7 (visible) and 0.8 (IR). Sol 314–315 continued to have substantial optical depth variability. The highest IR optical depths were near mid‐sol, but night optical depths rose during this period (Smith et al., 2022). During sols 313–315, sky brightness profiles in RDS also showed rapid variation, presumable to dust clouds moving through the fields of view. RDS‐measured total downwelling shortwave flux had a minimum on sols 316–317, >20% below nearby sols (imprecise due to saturated pre‐storm flux values near noon).

Sols 316–317 had some of the highest visible optical depths before the storm faded quickly on 318. However, during these sols, the optical depth variation settled into a morning to early afternoon peak with lower overnight values. Optical depth returned to ∼1, decreasing through sol 318. In the next sols, visible optical depths were near 0.4, with IR values about half that, and the seasonal trend of slowly rising optical depth resumed.

Dust in the lower atmosphere did not vary in synch with the column optical depths. A hill 36 km to the east was imaged and used to assess visibility and line‐of‐sight (LOS) opacity, sampling the lowest few km of the atmosphere. LOS opacity was computed from contrast following Moores et al. (2015). During the afternoon of sol 313, visibility across the crater dropped as low‐altitude dust increased. Through sol 315, LOS opacity varied with column optical depth, peaking at 1.3 ± 0.1 per 10.5 km when the hill was only faintly visible. On sols 316 and beyond, visibility was restored despite high column optical depths, with LOS opacity of ∼1 per 10.5 km (see Movie S1). By sol 319, the column optical depth and LOS opacity per scale height were again consistent. The implication is that for sols 313–315, the lower atmosphere filled proportionately with dust; while for sols 316–318, the rover experienced primarily high‐altitude dust from distant lifting areas.

### 3.3 Meteorological Measurements

Through sol 310, pressure amplitudes were representative of earlier sols. They increased in magnitude over sols 311–313 (Figure 1b). The maximum pressure range was ∼60 Pa on sol 313: this resulted from a diurnal‐amplitude increase from 10 to 23 Pa, a semi‐diurnal‐amplitude increase from 4 to 14 Pa, and high‐order terms (Sanchez‐Lavega et al., 2022a). Peak diurnal and semi‐diurnal amplitudes persisted during sols 313–315 during the period of low‐altitude dust and returned to pre‐storm values over the next 3 sols in the absence of low‐altitude dust. The diurnal tide's phase shifted 2.5 hr later between sols 311 and 313–315. The semi‐diurnal tide shifted later by 4 hr over sols 312–318, with a positive shift as long as the column amount of dust was high in the area.

Temperatures (Figure 1c) were impacted by the storm (Munguira et al., 2022) and the changing distribution of high‐versus low‐altitude dust. Over sols 312–315, the peak and average air temperature increased (by 12 and 5 K) with low‐altitude dust warming the daytime atmosphere, which was not observed by *Viking*, *Curiosity*, or *InSight*. By sol 316, the peak air temperature was cooler than typical, and the average was back to pre‐storm values, with compression of the diurnal range. The surface temperature had a lower maximum (10 K) and mean (4 K), but warmer minimum (6 K), for sols 316–317 due to reduced insolation and increased atmospheric thermal emission at night. Short timescale sonic temperature measurements (Chide et al., 2022) provided by the SuperCam microphone on sol 315 at 15:56 local mean solar time (LMST), show unusual temperature fluctuations of ±5.5 K/s compared to a mean value of ±3.2 K/s at this local time over the mission (see Figure S7 in Supporting Information S1).

Winds (Figure 1d) were only measured directly for a brief but eventful part of the storm. Pre‐storm winds were typically 4–8 m s^−1^ from the east during mid‐sol and 0–3 m s^−1^ from the east during night (Viudez‐Moreiras et al., 2022). High winds were seen during the vortex passage on sol 311 (Hueso et al., 2022). During the afternoon and evening of sol 312, winds shifted to 6 m s^−1^ southwesterly before becoming 4 m s^−1^ westerly. On sol 313 the wind vector rotated around the site clockwise, as winds became 5–10 m s^−1^ northwesterly and then northerly, with gusts above 20 m s^−1^, before becoming easterly at 10 m s^−1^. At 13:08, winds peaked at 22 m s^−1^ from 110° as a west‐moving vortex passed, and the wind sensor (WS) on boom‐2 stopped reporting due to damage from blown debris (see Figure S2 in Supporting Information S1). On sol 315, the other WS boom stopped reporting at 15:12. That time was coincident with a 200‐s optical depth spike that may have been due to local gusting.

Humidity played no role in the storm, but the 4–5 AM volume mixing ratio of water increased to a local maximum on sol 315. The reason is unknown, but the increased overnight temperature may have reduced adsorption into the regolith.

### 3.4 Local Dust Lifting

Vortices and dust lifting activity were common at the site (Newman et al., 2022) and persisted through the storm. Vortex activity was high through sols 313–315, and never unusually low (Hueso et al., 2022). Dust devils were imaged on sols 313 and 316–318 but were absent from MEDA data on sols 317–318. Dust devil motion from images was consistent with easterly or northeasterly mid‐sol winds on sols 316, 317, and 318.

**Figure 2:**
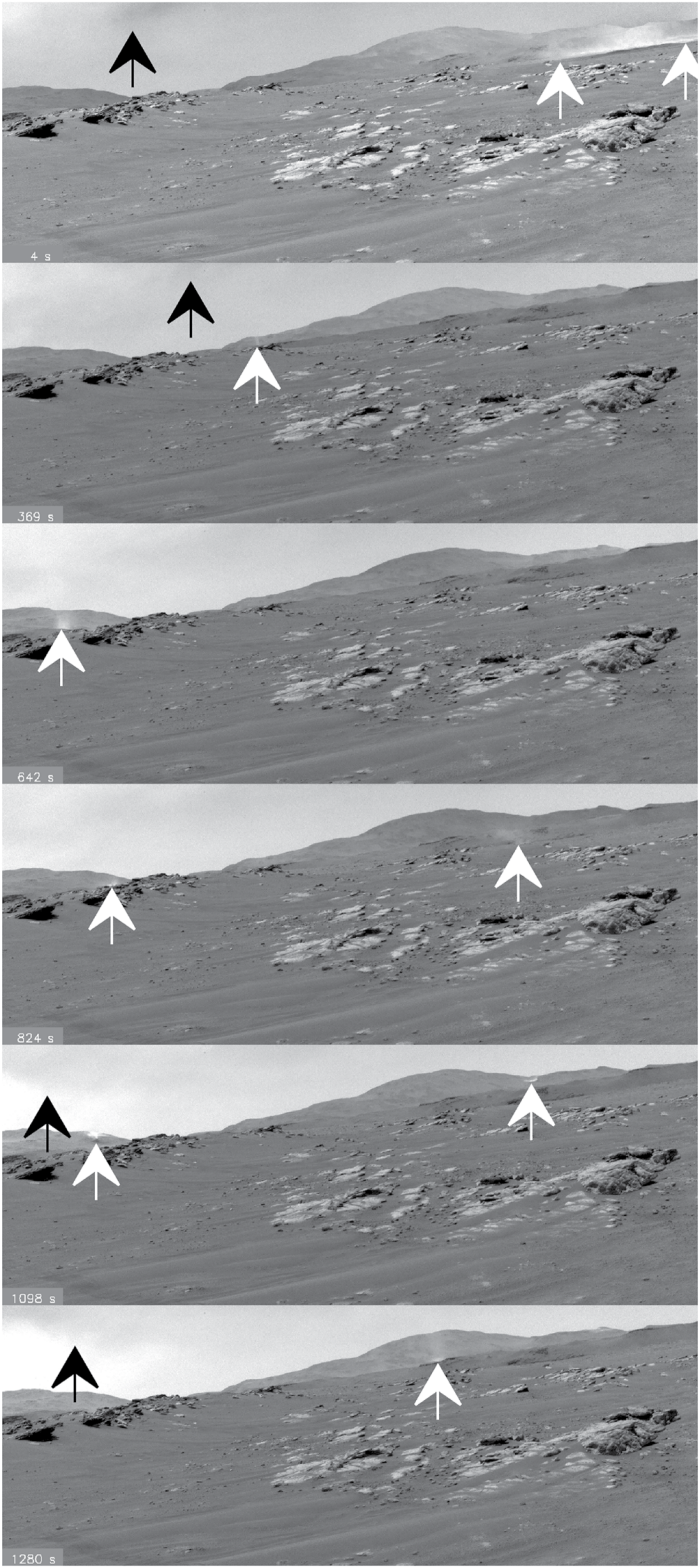
Dust activity seen in Navcam frames on sol 313 in sequence “ncam00535” looking toward azimuth 305°. Each image has been filtered via mean frame removal with 2x the difference frame added back in, to enhance visibility of the changes. (See Movies S2 and Movie S3).

Sol 313 had extensive dust lifting. Just before noon, the winds were ∼6 m/s from the north; imaging showed a mottled sky with rolling dust clouds, variable shadowing, and extreme dust lifting activity. Images (Figure 2) over 11:35‐11:59 showed 14 dust devils, more than any previous sol; three indeterminate dust lifting events; and four dust‐carrying gust events (cf., Newman et al., 2022). In late morning, RDS showed sky darkening in the north, followed by erratic brightness. The RDS showed 10% brightness variations over several‐minute periods from shadowing and local dust lifting. While dust devils moved southward near noon, images show high‐altitude dust clouds moved northward.

The dust devil activity early in the dust storm was different from that observed by Curiosity during the 2018 PEDE when strong suppression of vortex activity was observed (Ordoñez‐Etxeberria et al., 2020). However, the dust devil activity at Jezero diminished somewhat over the last few sols of the storm and recovered afterward (Hueso et al., 2022).

### 3.5 Storm‐Induced Changes

There were multiple instances in which winds from different directions, primarily east, were strong enough to mobilize sand and dust. Tracks and cuttings, not in equilibrium with the environment, were affected the most. Small‐scale ripples were mobilized, and local deflation and deposition occurred on natural surfaces and the rover.

**Figure 3:**
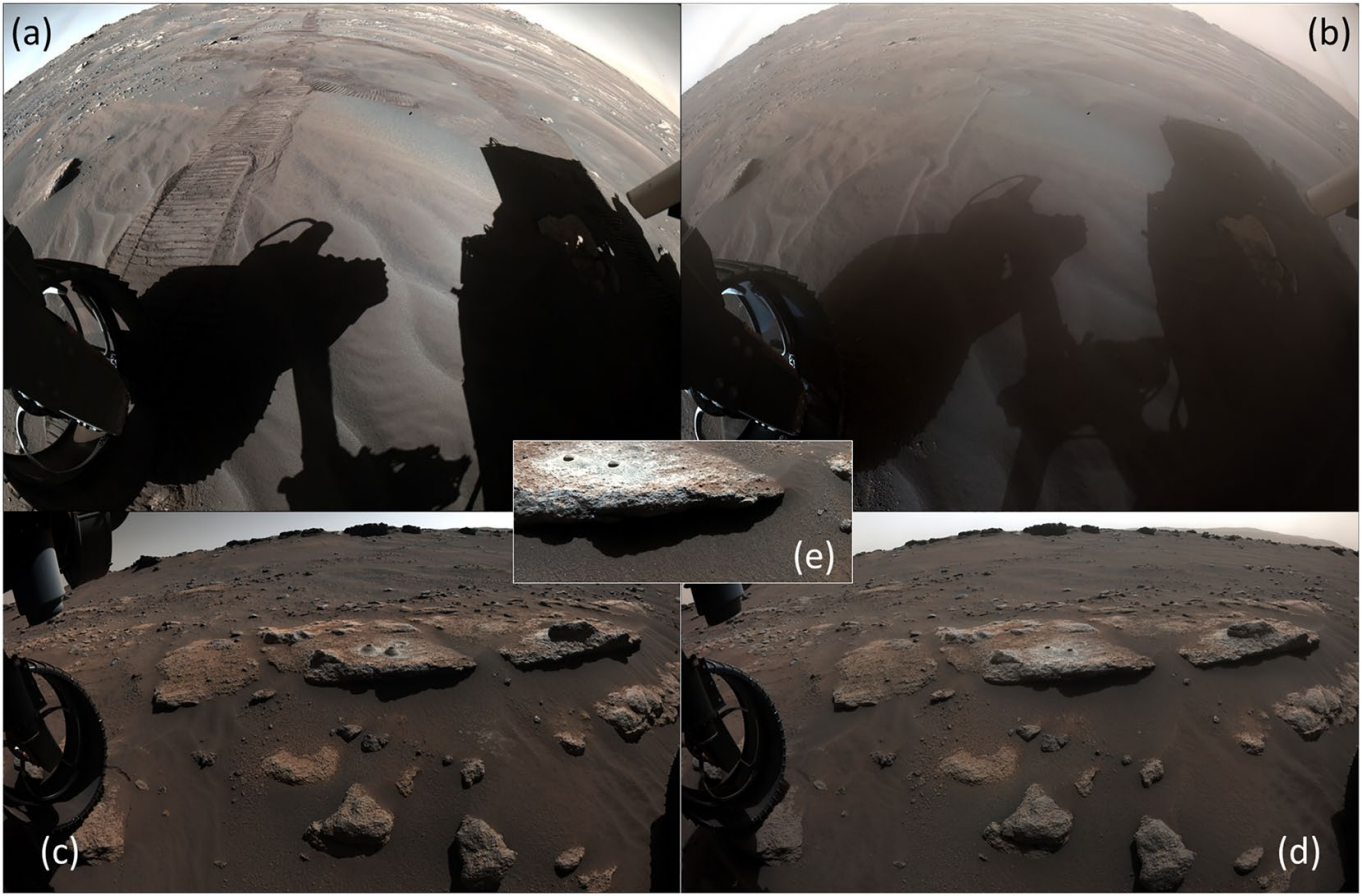
Changes immediately around the rover as seen in Rear (a), sol 286; (b), sol 316) and Front Hazcam (c), sol 311; (d), sol 315; (e), sol 322). (See Movie S3 and Movie S4.).

Sol 316 rear Hazcam images showed substantial modification and erasure of tracks to the east (Figure 3), and the optics became dust coated. Front Hazcam images on sol 314 showed removal of material from one abrasion site and two drill holes, as well as changes in other rover‐modified terrain, by winds from the east. Additional removal occurred between sols 314 and 315, and the cuttings were later scoured by winds from ENE before sol 320. Movement of mm‐sized grains was seen during RMI imaging on sol 315, associated with the large temperature fluctuations recorded by the SuperCam microphone.

**Figure 4:**
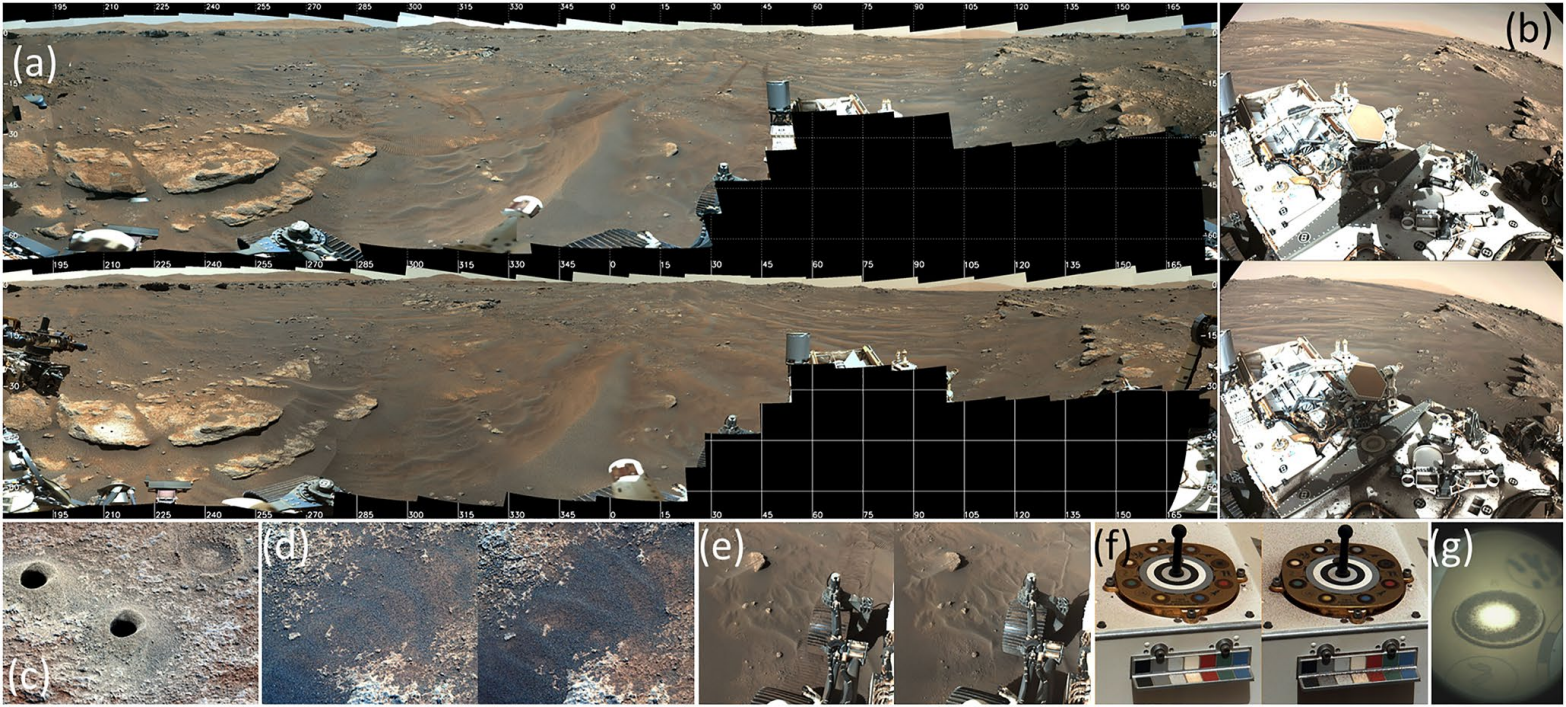
Changes in the rover site are shown in (a) sol 286–310 (top) and 321 (bottom) Mastcam‐Z panoramas; (b) Navcam deck images on sols 286 (top) and 321 (bottom); (c) sol 318 Mastcam‐Z 110‐mm image of drill cuttings removal; (d) Mastcam‐Z 110‐mm image of ripple migration from sol 290 (left) to 320 (right); (e) Navcam images showing tracks, ripples, and sand‐blasting of wheels, sol 286 (left) and 321 (right); (f) Mastcam‐Z calibration target images; and (g) a calibration target detail with Supercam (Maurice et al., 2021). (See Movies S5–S10).

Site imaging after the storm (Figure 4) showed a series of changes. (a) The rover itself was affected. The deck (∼1 m high) had a substantial increase in its sand load (some of which was last modified by winds from the west, based on piling against obstacles). Wheels were scoured clean. Clods (or large grains or aggregates) were removed from the deck near the Mastcam‐Z reflectance calibration target (RCT) on sol 311–312 and 314–315. Over 315–316 there was a debris dump on the targets, but material was removed from magnets by winds from the SE (Figure 4g). (b) Rover‐modified terrain was substantially reworked. Tracks to the east, south, and west were impacted. Some tracks disappeared; others were smoothed by motion of sand. (c) Small ripples all around the rover moved westward, and sand moved on rocks.

Albedo was monitored by MEDA's RDS and TIRS in the near‐field near azimuth 300 in Figure 4 (Martínez et al., 2022; Vicente‐Retortillo et al., 2022). Albedo decreased 11% from sol 315 to 316 and ∼6% more by sol 319, possibly due to dust removal.

The minimum set of sediment mobilization events during sols 311–318 includes one minor pre‐storm event and three in‐storm events: (a) 311M11:44 (i.e., 11:44 LMST on sol 311); (b) 313M13:08; (c) 314M12:53–315M12:10; and (d) 315M15:12 and shortly thereafter. The first event was not associated with the storm, but was a vortex passage: a 4.8‐Pa pressure drop with wind gusts to >14 m s^−1^. Simultaneous surface dust removal was inferred from a surface albedo decrease (Vicente‐Retortillo et al., 2022), and this was considered the most likely time for the first clod removal from the RCT (311M11:10–312M15:08). Notably, the vortex occurred prior to the final image of the undisturbed workspace by the front Hazcam, and no other >1‐Pa vortices were measured until sol 313. The second event was the WS2 failure; the first workspace changes as well as the east and north track modification may have been associated with this event. The third range was the time interval during which the second RCT clod removal and second workspace changes occurred; these also partially overlapped the time during which track modification occurred. The final event was the WS1 failure; this overlaps the final workspace scouring, the sediment deposition on the RCT, and the possible times for east track modification. In addition, mm‐sized grain motion was imaged by RMI shortly after the WS1 failure (see Movie S11). It is possible there were more events—it is likely that more than one event mobilized the disturbed material in rover tracks. However, it is likely that—given the large change to the cuttings and the deposition of material onto the rover deck—the sediment coverage of the rover deck is at least partly associated with at least the final event. (Additional time constraints appear in Text S1 in Supporting Information S1).

## 4 Conclusions

A significant dust storm impacted the *Perseverance* site for six sols, starting on sol 313 (L_S_ 153°). The leading edge of the storm brought a dynamic local environment that resulted in increased dust lifting and sediment mobilization, unlike prior in situ storm encounters (Guzewich et al., 2019; M. Lemmon et al., 2015; Ryan & Henry, 1979). During the first sol, winds rotated around the area clockwise, increased in speed, and drove dust devils and dusty gusts. At least three sediment‐mobilization events occurred, resulting in extensive reworking of rover‐modified terrain, sand and ripple migration, damage to the wind sensor, coating of cameras, and deposition of sediment on the rover deck. No winds >24 m/s were measured, but larger winds may have occurred after the loss of the wind sensor. Wind shear was observed in the first sol, with surface‐level northerly winds measured locally and observed via dust devil motion, while dust clouds in the sky were driven by southerly winds.

Optical depth was high and variable through the storm, and line‐of‐sight visibility initially fell due to low‐altitude dust. Low visibility persisted for three sols, but visibility was restored during sols 316–318, marking a reduction in low‐level dust even while the column dust amount peaked. The reduction in low‐level dust may have been associated with a less dynamic local atmosphere, with no mobilization events known to have occurred after sol 315; dust devils were imaged but reduced in frequency in the last 3 sols. There were sky brightness variations in the first three sols, presumably due to variability in low‐altitude dust. Dust variability occurred over all timescales on the first three sols but settled into a diurnal pattern with a mid‐sol peak and an overnight minimum over the last sols.

Pressures and temperatures responded to both the dust column and the vertical distribution of the dust. Average and peak atmospheric temperatures were elevated during sols with low‐altitude dust and local dust lifting, unlike prior in situ storm encounters (Guzewich et al., 2019; Ryan & Henry, 1979; Viúdez‐Moreiras et al., 2020). Average and peak surface temperatures decreased, and minimum temperatures increased, during the highest column optical depths, as at other sites. The amplitude of pressure variation increased, and its phase shifted later, due to the enhanced dust (as in Tillman, 1988; Viúdez‐Moreiras et al., 2019, 2020; Zurita‐Zurita et al., 2022). The pressure amplitude maximum occurred during the maximum dust loading of the lower atmosphere, as did the peak phase shift of the diurnal tide. The phase of the semi‐diurnal tide continued to shift while dust was elevated, before returning to pre‐storm conditions as the storm abated.

## Conflict of Interest

The authors declare no conflicts of interest relevant to this study.

## Supporting information

Supporting Information S1

Movie S1

Movie S2

Movie S3

Movie S4

Movie S5

Movie S6

Movie S7

Movie S8

Movie S9

Movie S10

Movie S11

## Data Availability

All Perseverance data used in this study are publicly available via the Planetary Data System (J. F. Bell and Maki, 2021; J. M. Maki, 2021; Rodriguez‐Manfredi and de la Torre Juarez, 2021; Wiens & Maurice, 2021).
