# Supplementary material for: Dust, Sand, and Winds Within an Active Martian Storm in Jezero Crater
Source: Geophys Res Lett. 2022 Sep 9;49(17):e2022GL100126. doi: 10.1029/2022GL100126 (PMC9540647; doi:10.1029/2022GL100126)
Supplement: Supplementary file 1 — Supporting Information S1 [file GRL-49-e2022GL100126-s009.docx]

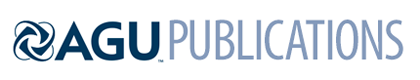


*Geophys. Res. Lett.*

Supporting Information for

**Dust, Sand, and Winds within an Active Martian Storm in Jezero Crater**

M.T. Lemmon^1^, M.D. Smith^2^, D. Viudez-Moreiras^3^, M. de la Torre-Juarez^4^, A. Vicente-Retortillo^3^, A. Munguira^5^, A. Sanchez-Lavega^5^, R. Hueso^5^, G. Martinez^6^, B. Chide^7^, R. Sullivan^8^, D. Toledo^9^, L. Tamppari^4^, T. Bertrand^10^, J.F. Bell III^11^, C. Newman^12^, M. Baker^13^, D. Banfield^8^, J.A. Rodriguez-Manfredi^3^, J.N. Maki^4^, V. Apestigue^9^

^1^Space Science Institute, Boulder, CO 80301, USA. ^2^NASA Goddard Space Flight Center, Greenbelt, MD, USA. ^3^Centro de Astrobiologia (INTA-CSIC), Madrid, Spain. ^4^Jet Propulsion Laboratory, California Institute of Technology, Pasadena, CA. ^5^Física Aplicada, Escuela de Ingeniería de Bilbao, UPV/EHU, Bilbao, Spain. ^6^Lunar and Planetary Institute, Houston, TX, USA. ^7^Space and Planetary Exploration Team, Los Alamos National Laboratory, Los Alamos, New Mexico, USA. ^8^Cornell Univ., Ithaca, NY, USA. ^9^Instituto Nacional de Técnica Aerospacial, Madrid, Spain. ^10^LESIA, Observatoire de Paris, Meudon, France.^11^Arizona State University, Tempe, AZ, USA. ^12^Aeolis Research, Chandler, AZ, USA. ^13^Smithsonian National Air and Space Museum, Washington, DC, USA.

**Contents of this file**

Text S1 to S5

Figures S1 to S8

**Additional Supporting Information (Files uploaded separately)**

Captions for Movies S1 to S11

**Introduction**

Text S1 and Fig. S1 illustrate the progression of the storm via orbital imagery. Text S2 and Fig. S2-S3 illustrating winds and vortices in the storm. Text S3 and Figs. S4-S5 describe the processing of dust lifting movies. Text S4, Fig. S6-S7, and Movies S1-S11 illustrated the timing and nature of changes at the landing site through the storm. Text S5 and Fig. S8 illustrate the variation of downwelling solar and thermal irradiances during the storm.

Text S1. Progression of the storm.

Figure S1 shows orbital images taken from projections of global maps. The Perseverance site was imaged in mid-afternoon each sol (except sol 319). All processing was done by Malin and Cantor, 2022a; b. The white circle represents the position of *Perseverance*.

At the site, Navcam images of the eastern sky also show a 36-km distant hill (Movie S1). Dust clouds can be seen in the early frames. The visibility to the hill was determined using methods from Moores et al., (21015). The near-field ground was used as an analog for the distant surface brightness; the brightness of the distant feature was measured; and the brightness of the sky to either side of the feature was measured. The optical depth to the feature was computed using equation 22 from Moores et al. (2015). That was converted to a number that could be compared with column optical depth by scaling to 10.5 km from 36 km.


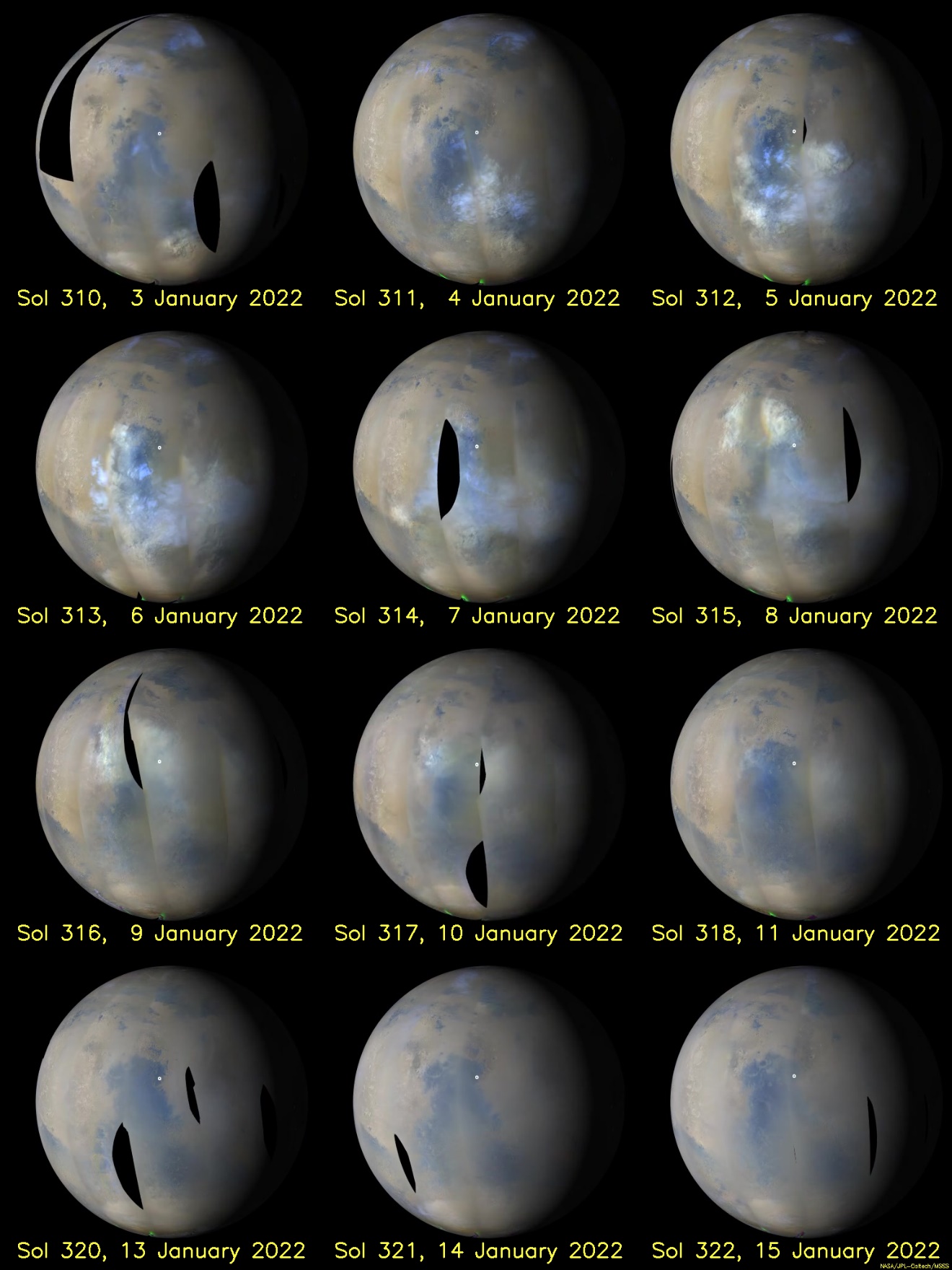
Figure S1. Progression of the storm from Mars Color Imager (MARCI) images. Projections of the MARCI weather report images center on the *Perseverance* longitude are shown for 3-15 January (omitting 12 January due to lack of coverage). The top row shows the sols prior to the storm’s impact at Jezero; the next row shows the three dynamic sols; the next row shows the three dusty sols; the bottom row shows three post-storm sols. All images credit: NASA/JPL-Caltech/Malin Space Science Systems (MSSS). Public domain images from 3-10 January were created and published by Malin and Cantor., 2022a (captioned image release No. MSSS-604); those from 11-15 January were created and published by Malin and Cantor, 2022b (captioned image release No. MSSS-605).

Text S2. Winds and vortices.

To facilitate interpretation of wind changes, winds were averaged over 15-minute intervals and shown in Fig. S2 in hodograph form. Light blue colored points represent mid-sol easterly (wind vector to west) winds. Dark purple-brown points represent overnight slow westerlies. Yellow-green-cyan points show the morning evolution through low winds from westerly to easterly. Violet-purple points show evening winds rotating around to weak northerlies. The afternoon of sol 312 (e) shows an unusual southwesterly; sol 313 (f) shows a mid-sol northerly wind that rotated around to easterly prior to loss of the wind sensor.

Vortices were determined from pressure drops and classified as dusty or not based on RDS Top-7 fluxes. Vortex and dust devil frequency through the storm are in Fig S3.


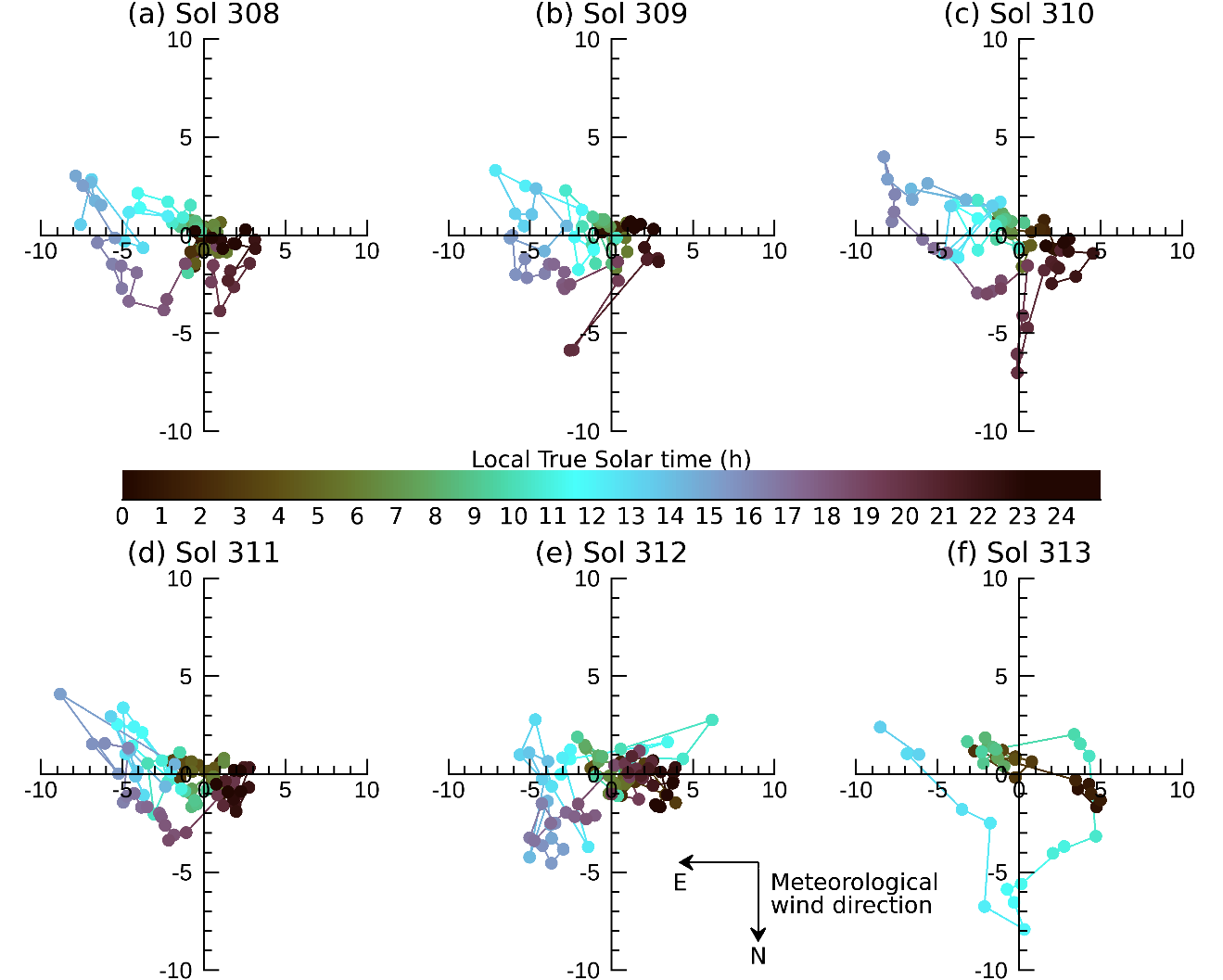
Figure S2. Wind hodograph for sols 308-313. Color indicates local true solar time, with lighter colors indicating daytime and darker colors indicating night. The 15-minute average wind vector at any time is from the origin to the point. The meteorological wind direction is shown (i.e., easterly winds come from the east and the vector points to the west).


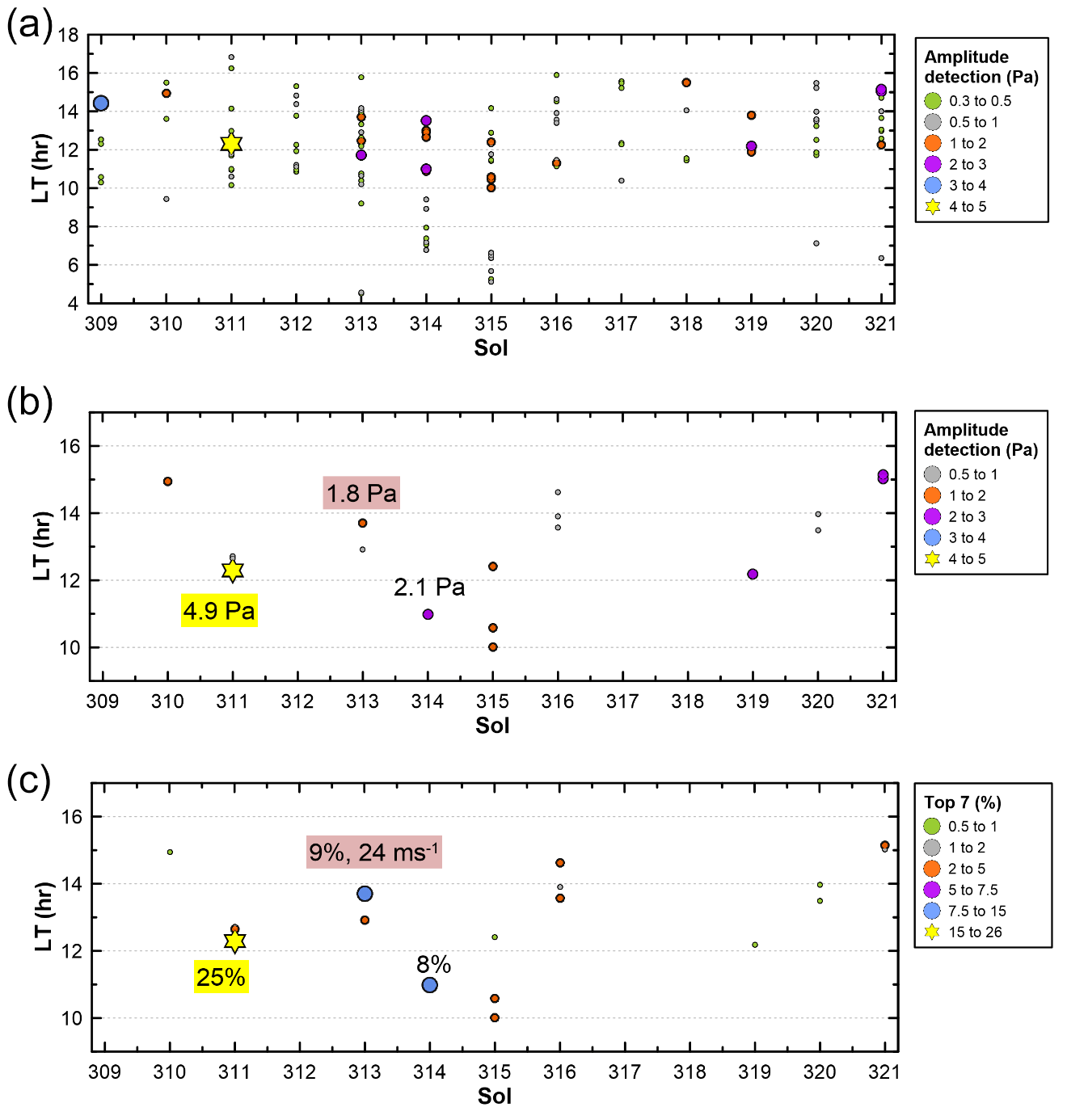


Figure S3. Vortex and dust devil activity over the dust storm from MEDA pressure and TOP7 data. (a) Convective vortices with pressure drops with maximum ΔP drops from 0.3 to 4.9 Pa. (b) Convective vortices with additional response in the TOP7 downward shortwave flux data indicating the close passage of a dust devil and highlighting the maximum ΔP measured. (c) Same as (b) but highlighting the maximum drop of irradiance measured with the TOP7 detector. There are three events highlighted on sols 311, 313 and 314. The event on sol 311 was exceptional in terms of its very strong signal in the TOP7 sensor. The event on sol 313 was conventional in its pressure drop, but extremely dusty for an event of that pressure intensity and accompanied by very strong winds and was responsible of the damage to the wind sensor. The event on sol 314 was an event comparable in magnitude and characteristics without producing additional effects.

Text S3. Dust lifting movie processing.

Dust lifting movies discussed and shown in the main text were processed using methods like those in Greeley et al. (2010). For sol 313, sequence ncam00535, 45 images were taken as 15 sets of three images. Two of the three images in each set were down-sampled (DS) two times. They were rescaled to full size by taking the ratio of each DS image to a DS version of the full-sized image, rescaling the ratio with bi-linear interpolation, and multiplying the result back into the full-sized image. The technique preserved high-frequency information from the scene. Then, the 45 images were averaged with minimum and maximum point rejection for each pixel. The mean frame is shown in Fig. S4. Difference images (Movie S2) were computed by subtraction of the mean frame. Enhanced images (Movie S3) were computed by adding twice the difference images to the original images (i.e., tripling the contrast of changing features).

A dust survey occurred just before the movie (above) and showed distant dust devil and a dust-laden wind gust (Fig. S5). The survey comprised 5 aims with three images each. For each aim, the left column shows the mean frame (monochrome) with the three difference frames added back in to the red, green, and blue channels (such that a bright feature’s motion can be seen as a red-green-blue sequence). The right column shows the ratio of the first and last frame for each aim. (The arc in the first frame is a common artifact.)


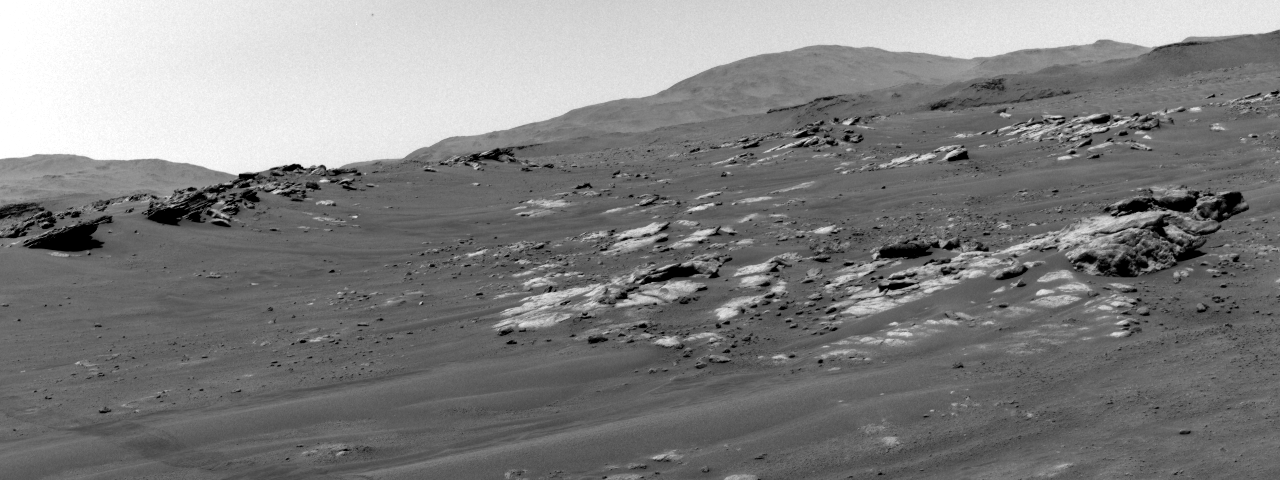
Figure S4. Mean from for dust lifting movie from sol 313, sequence ncam00535 at 11:04 LMST (11:38 LTST).


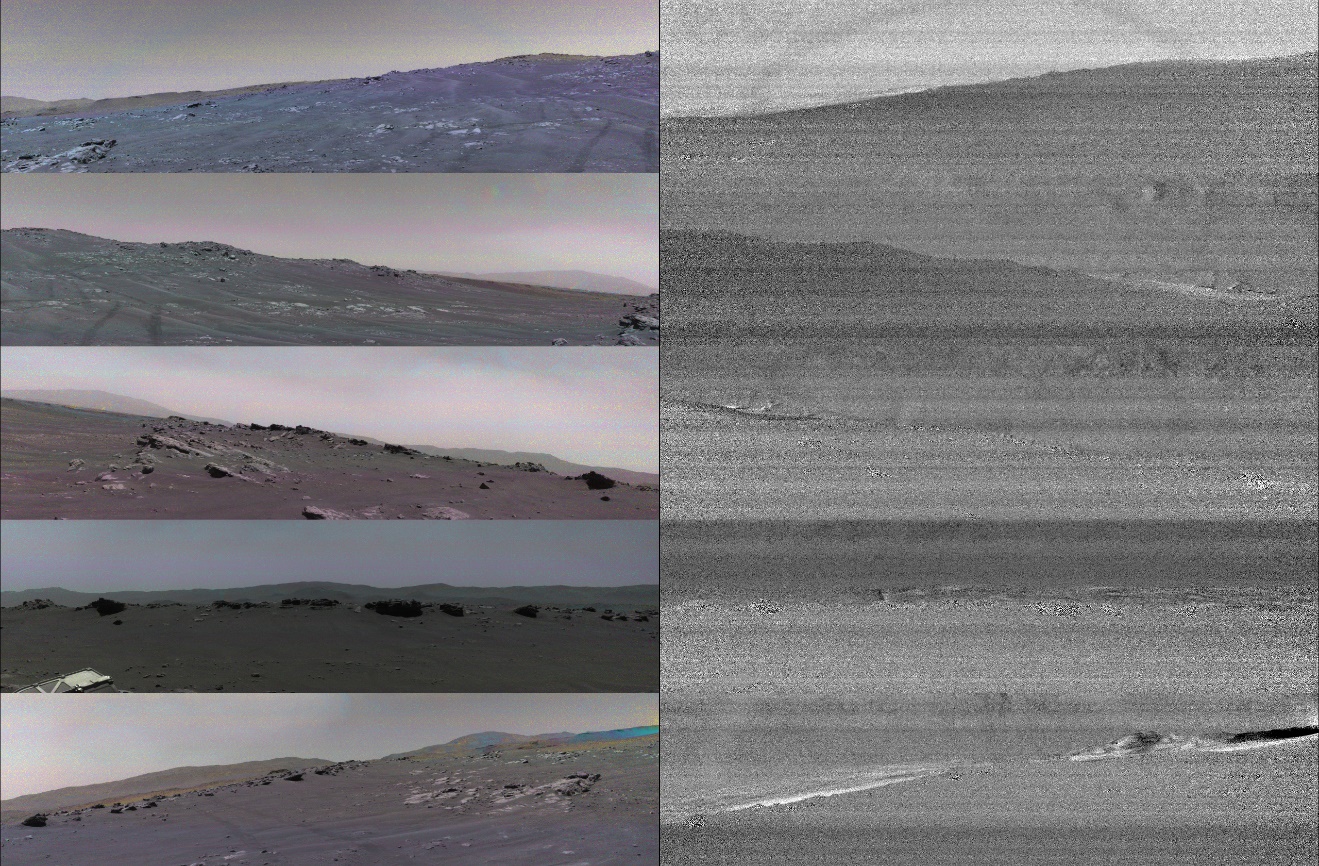
Figure S5. Mean from for dust lifting survey from sol 313, sequence “ncam00500” at 11:04 LMST.

Text S4. Change detection.

Here and in Fig. S6, we summarize key observation used to determine when changes occurred and the nature of the changes that happened. This section discusses a subset of observed changes.

Rear: Terrain modification behind rover and RHAZ coating happened before **316M14:54** (~easterly). See movie S4.

Workspace and cuttings: There were at least three instances of substantial movement of material. Some terrain modification in front of the rover (fissure, light-toned debris) happened before **314M12:53**. Drill cuttings were modified before **314M12:53** and *much* more between **314M12:53** and **315M13:06** (easterly wind); the cuttings were later scoured by winds from ENE before sol **320**. See movie S5.

Tracks: Comparison of dust devil survey images constrained major NE track modification to between **313M11:01** and **315M12:14**. Cloud survey images showed track modification to between **313M15:10** and **314M15:35**, with some likely after that sol 314 time. Mastcam-Z images show tracks to the north of the rover were modified by 315M12:03, although additional modification is not excluded.

Wind sensor (WS): Damage occurred at sols **313M13:08** and **315M15:12**.

Reflectance calibration target (RCT): ZCAM cal target lost clods over **311-312** (likely vortex-related) and **314-315**; and gained substantial debris while losing material from magnets in southeasterly wind over **315-316** (all near 11-12 LMST). See movie S6.

Sometime during the storm: The deck gained debris in unknown winds; the last motion of some (not all) material was to the east, based on where it piled (once on deck, it became easier to move). Wheels were scoured clean. Smallest-scale ripples migrated generally to W. See movies S7-10.

Chandon: A target associated with rover tracks was imaged and interrogated with SuperCam. Movie S11 shows the overlap of RMI images illustrating grain motion, while Fig. S7 shows unusual temperature fluctuations.

The SuperCam microphone (Maurice et al. 2022) enables the retrieval of the near-surface atmospheric temperature and its fluctuation, at an unprecedented timescale, using the dependence of sound speed on temperature (see Chide et al. this issue for the method and a detailed review of the results). The sound speed derived air temperature, hereafter the sonic temperature, is computed for every SuperCam laser shots fired on each rock targets. This acoustic based technique allows the retrieval of intense and short time scale temperature fluctuations due to its fast response time of about 10ms. Sonic temperature fluctuations are shown to follow the diurnal pattern of the turbulence (Fig. S6). Measurements acquired on target Chandon on sol 315 at 16:08-16:30 LTST show an unusual magnitude of the temperature fluctuations at this local time, likely due to an increased turbulence linked with the passage of the storm. In addition, two other targets were recorded during the dust storm: Riolan on sol 312 and Tanaron on sol 313. Although Tanaron does not show any unusual temperature fluctuation (±3.3 K/s), Riolan shows fluctuation between 4.6K/s and 6.2K/s, which is higher than the average value at this local time.


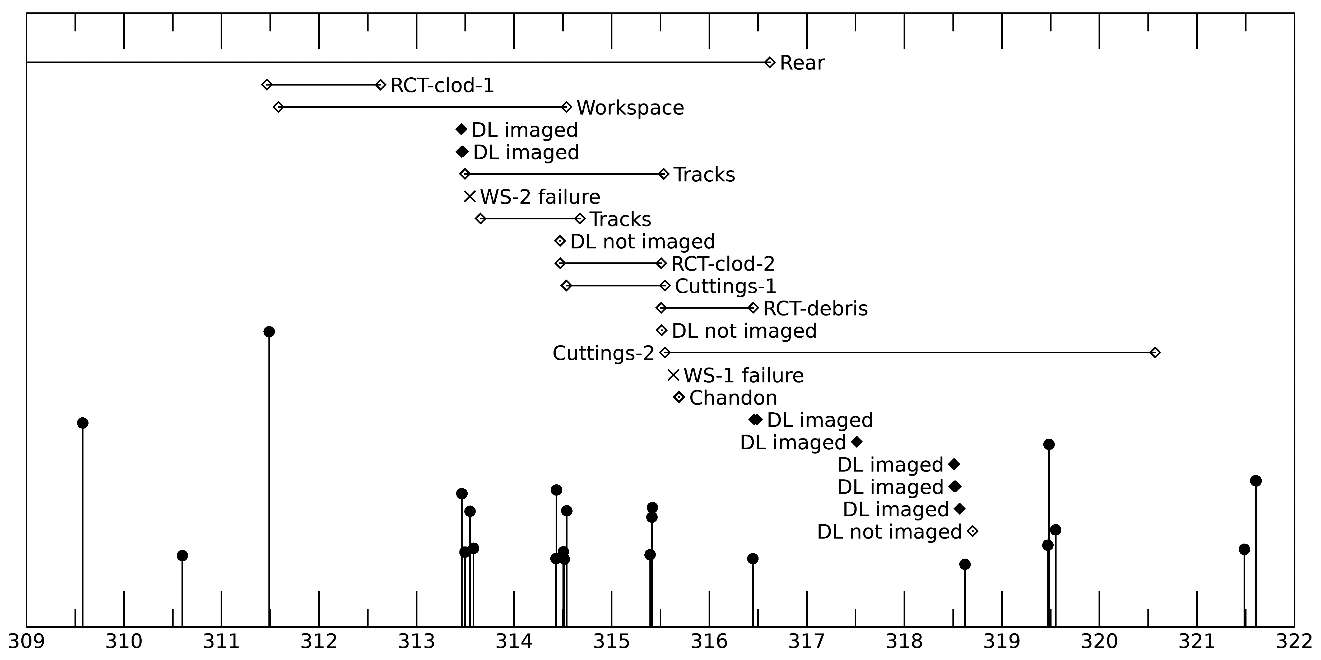


Figure S6. Timeline of removal events. Time constraints for select changes discussed in the text is shown. Imaging sequences are coded as to whether dust lifting (DL) was imaged (filled diamonds) or not imaged (open diamonds). Pressure drops of <120 s duration and >1 Pa change are shown (line and filled circle); the full height of the plot corresponds to a 10-Pa drop.


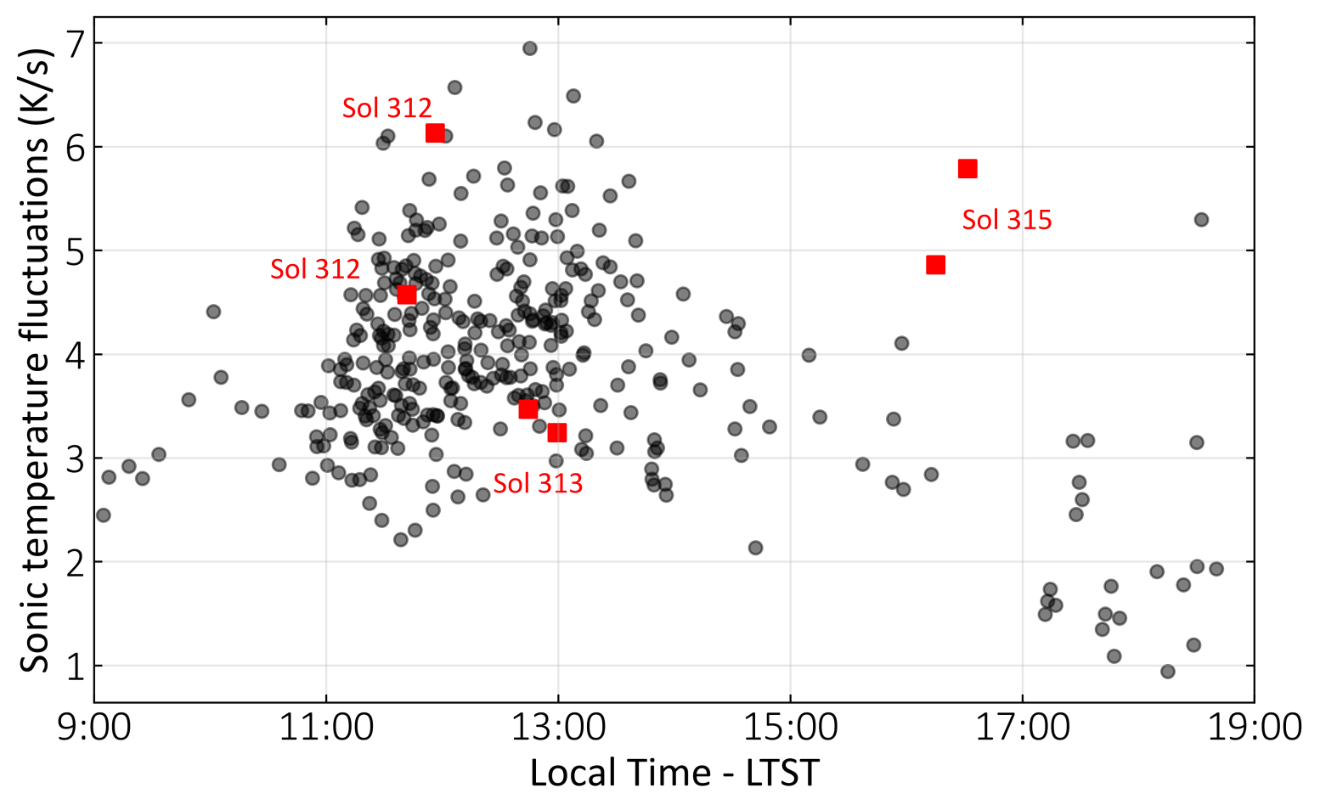


Figure S7. Diurnal evolution of the temperature fluctuations recorded with acoustics (black). The three targets sampled during the dust storm are highlighted in red squares. There are two points per targets as the fluctuations are computed over 150 shots and these targets were sampled with 300 shots.

Text S5. Fluxes.

The downwelling shortwave flux (RDS Top-7) was reduced during the storm in response to the visible optical depth (Fig. S8). The downwelling thermal flux (TIRS) increased during the day and night, with both sol-to-sol and high-frequency changes related to changing dust amounts, and diurnal and sol-to-sol changes related to changing temperature.


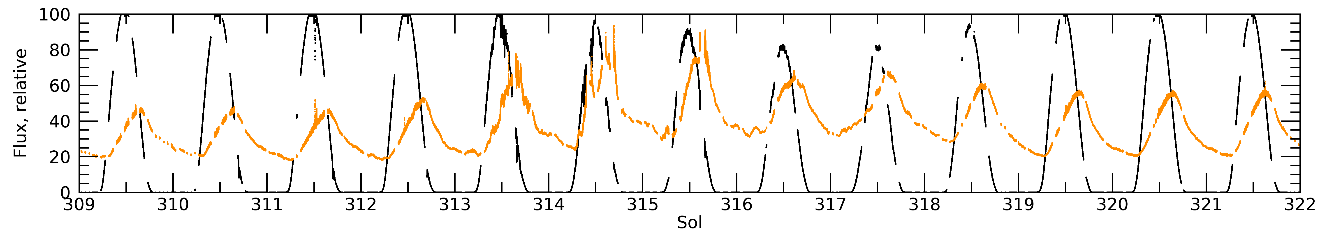


Figure S8. Dowelling shortwave (black) and thermal (orange) irradiance from RDS and TIRS, respectively. The RDS Top-7 was saturated at midsol outside of the storm. The scaling is arbitrary (100% is 3.6 W m^-2^ over 0.19-1.1 µm and 0.5 W m^-2^ over 6.5-30 µm for Top-7 and TIRS, respectively)

**Additional Supporting Information (Files uploaded separately)**

Movie S1. Animation of 8 Navcam frames from sequence “ncam00501” overs sols 312-321 looking above the horizon to the southeast. Dust clouds can be seen in the sky, and the 36-km distant feature used to track visibility can be seen in the lower left.

Movie S2. Animation of 45 Navcam difference frames from sequence “ncam00535” on sol 313 around 11:04-11:25 LMST looking 55° west of north (azimuth 305°). The images are computed as the difference between individual frames and the mean frame, and thus highlight areas that change.

Movie S3. Animation of 45 Navcam enhanced frames from sequence “ncam00535” on sol 313 around 11:04-11:25 LMST looking 55° west of north (azimuth 305°). The images are computed as the sum of the original frame and two times the difference frames, thus illustrating the areas dust lifting is seen by enhancing its visibility.

Movie S4. Animation of two Left Rear Hazcam frames from sols 286M14:28 and 316M14:54. The comparison shows track erasure and a contrast-reducing coating on the Hazcam optics that also appeared on the Right Rear Hazcam.

Movie S5. Animation of Left Front Hazcam frames from sols 311M14:00, 314M12:53, 315M13:06, and a composite of images from 320M13:44 and 322M12:59. The composite was required due to saturation in the sol 320 image and a small subframe for the sol 322 image. The comparison shows changes to the workspace and drill cuttings and abrasion site.

Movie S6. Animation of nine Mastcam-Z images of the RCT from sols 309-319. Key images were at 311M11:10, 312M15:08, 314M11:20, 315M12:10, and 316M10:48 (as the rover was stationary, the shadow indicates local time for other images). The comparison shows two clod removal events from the deck around the RCT and a substantial debris event that also resulted in southeasterly removal of material from the magnets surrounding the sun-dial.

Movie S7. Animation of two Navcam frames from the sol 286 and 321 site panoramas. The comparison shows sediment deposition on the deck.

Movie S8. Animation of two Navcam frames from the sol 286 and 321 site panoramas. The comparison shows sediment deposition on the deck.

Movie S9. Animation of two Navcam frames from the sol 286 and 321 site panoramas. The comparison shows track erasure, wheel cleaning, and eastward (to left) ripple migration.

Movie S10. Animation of two Mastcam-Z frames from the sol 290 and 320 site panoramas. The comparison shows eastward (to upper right) ripple migration.

Movie S11. Animation of two RMI frames from the sol 315 masked to show only overlapping areas. A 1-mm scale bar appears in lower left.
