# Supplementary figures and images for: Dust, Sand, and Winds Within an Active Martian Storm in Jezero Crater
Source: Geophys Res Lett. 2022 Sep 9;49(17):e2022GL100126. doi: 10.1029/2022GL100126 (PMC9540647; doi:10.1029/2022GL100126)

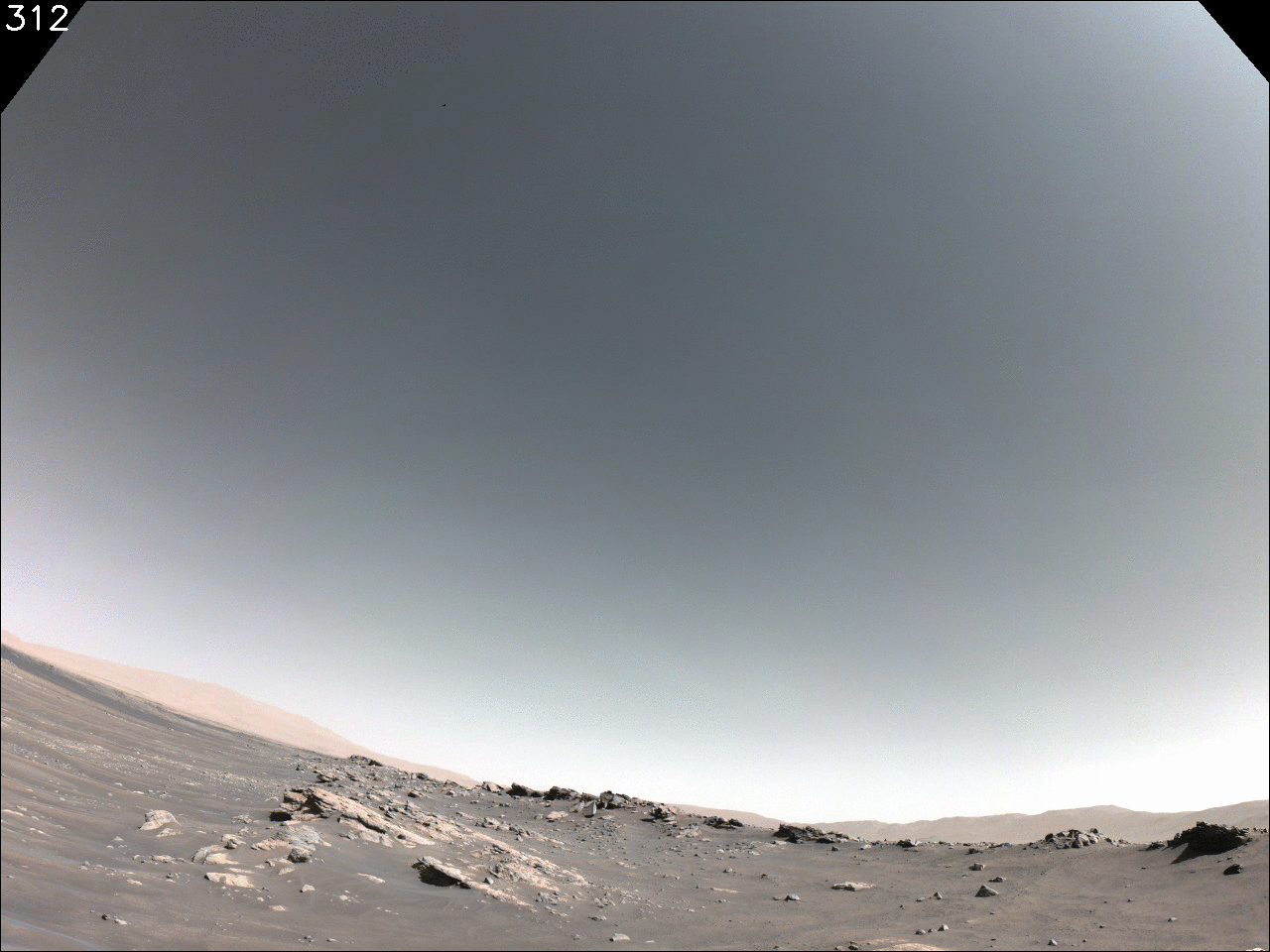

Supplement: Supplementary file 2 — Movie S1 [file GRL-49-e2022GL100126-s011.gif]

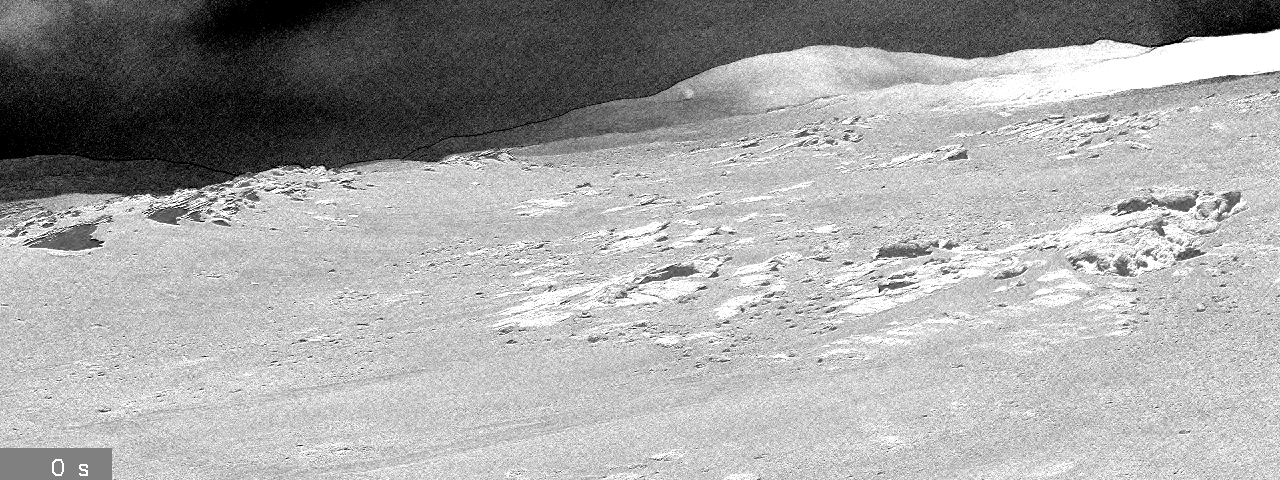

Supplement: Supplementary file 3 — Movie S2 [file GRL-49-e2022GL100126-s008.gif]

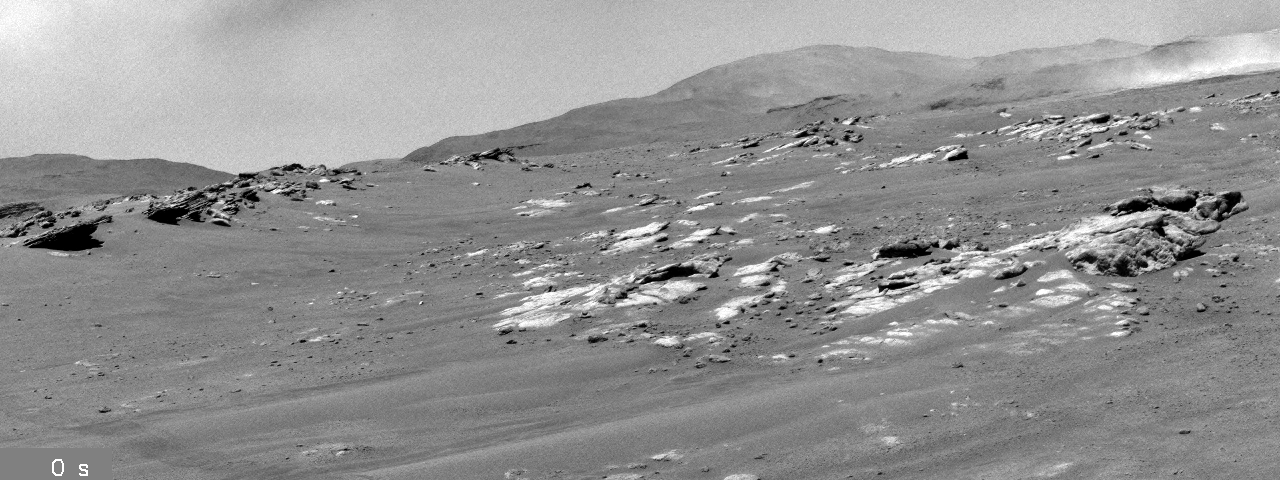

Supplement: Supplementary file 4 — Movie S3 [file GRL-49-e2022GL100126-s010.gif]

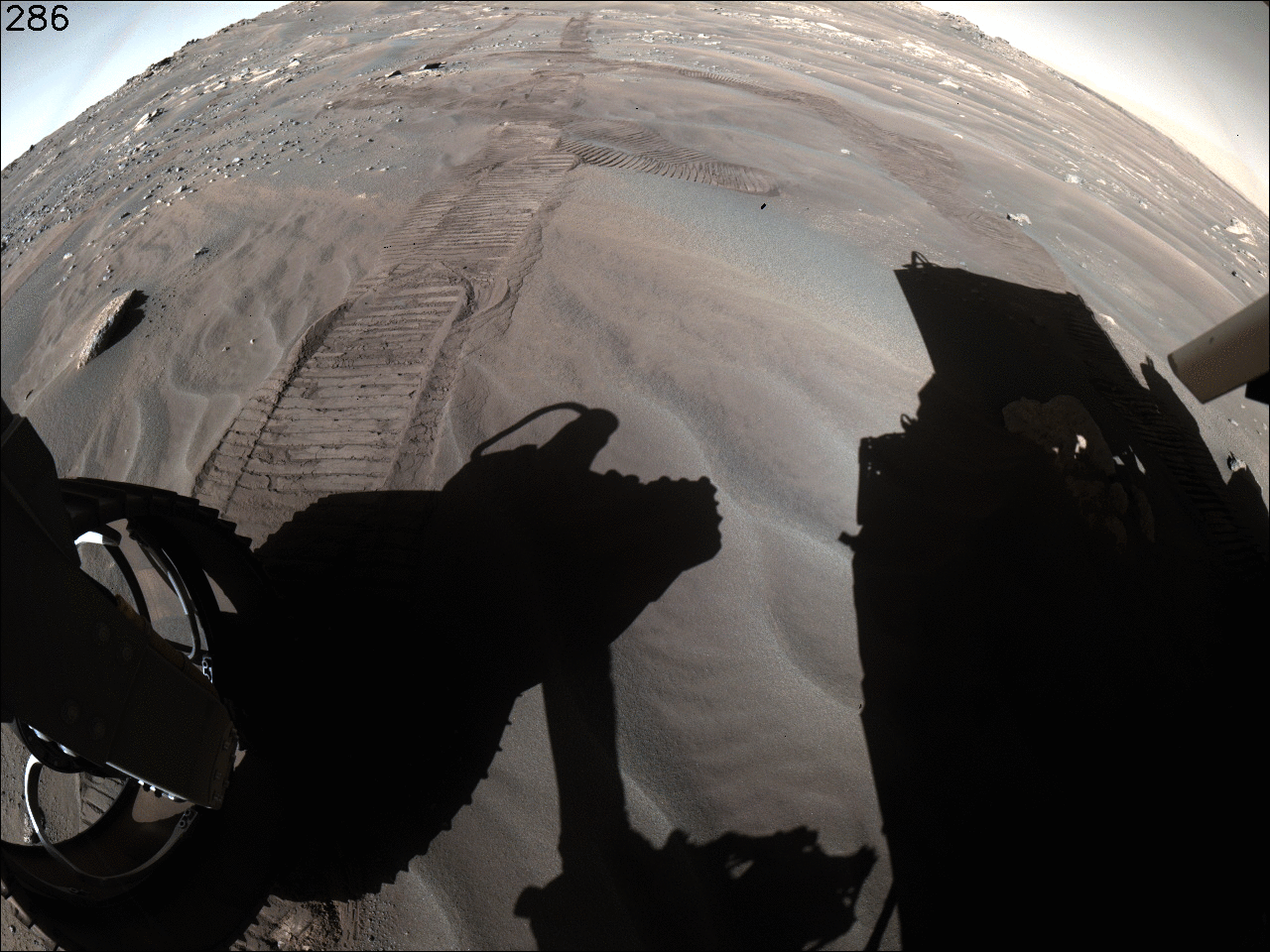

Supplement: Supplementary file 5 — Movie S4 [file GRL-49-e2022GL100126-s004.gif]

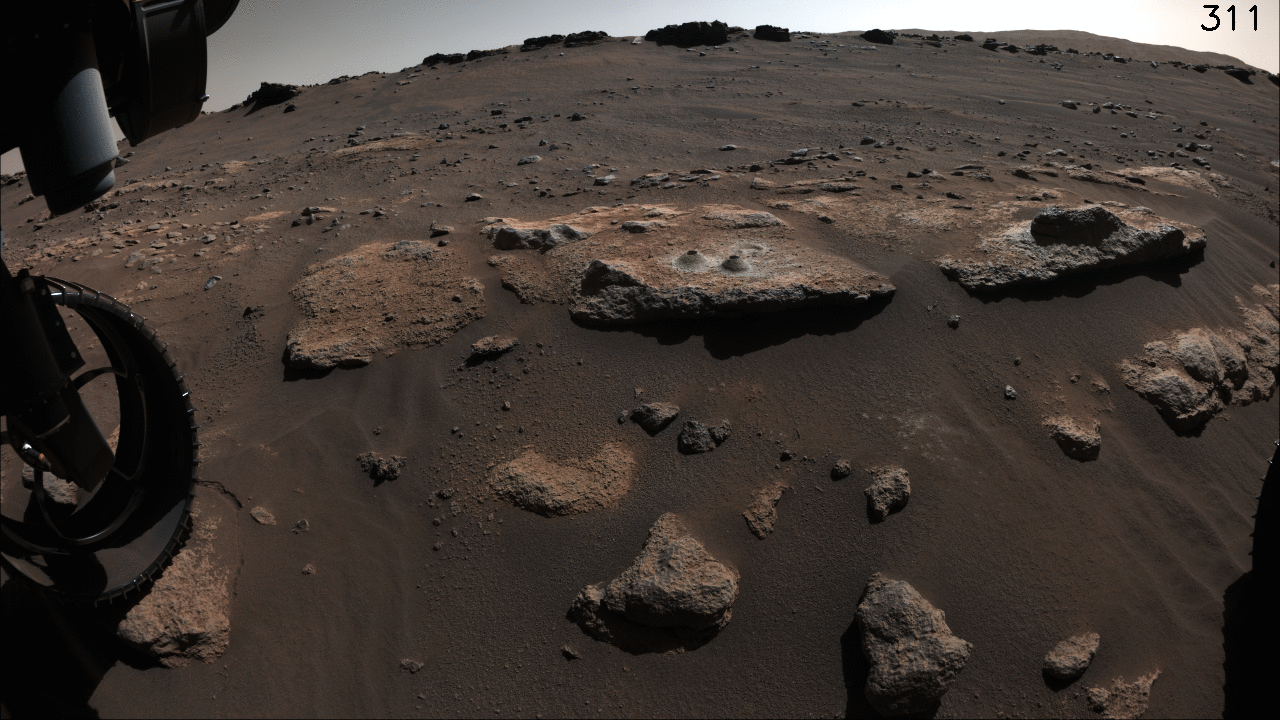

Supplement: Supplementary file 6 — Movie S5 [file GRL-49-e2022GL100126-s012.gif]

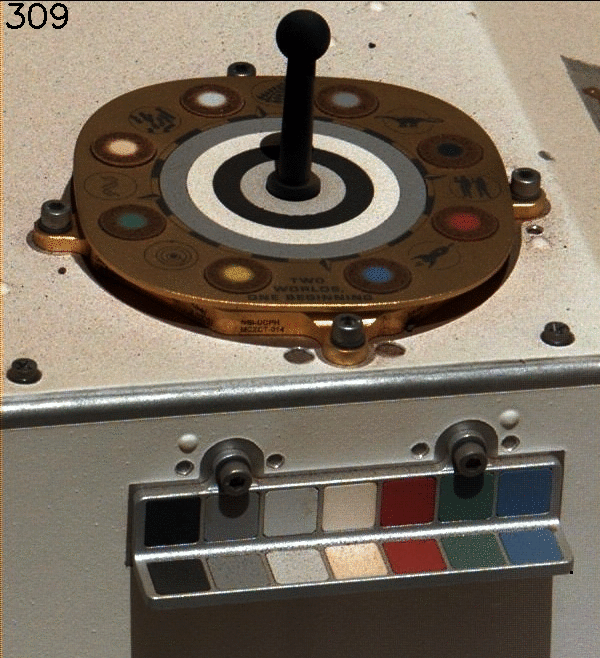

Supplement: Supplementary file 7 — Movie S6 [file GRL-49-e2022GL100126-s007.gif]

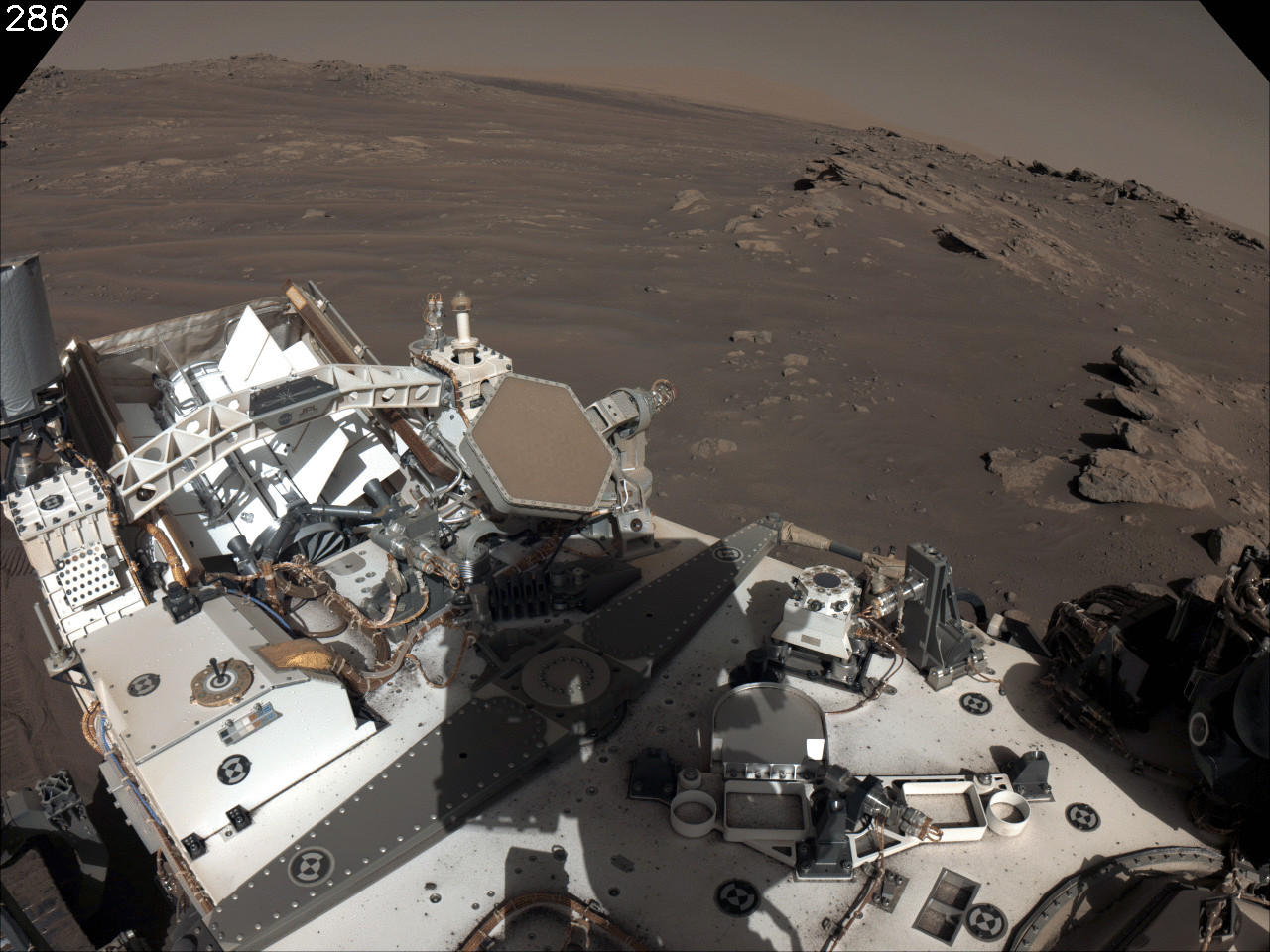

Supplement: Supplementary file 8 — Movie S7 [file GRL-49-e2022GL100126-s001.gif]

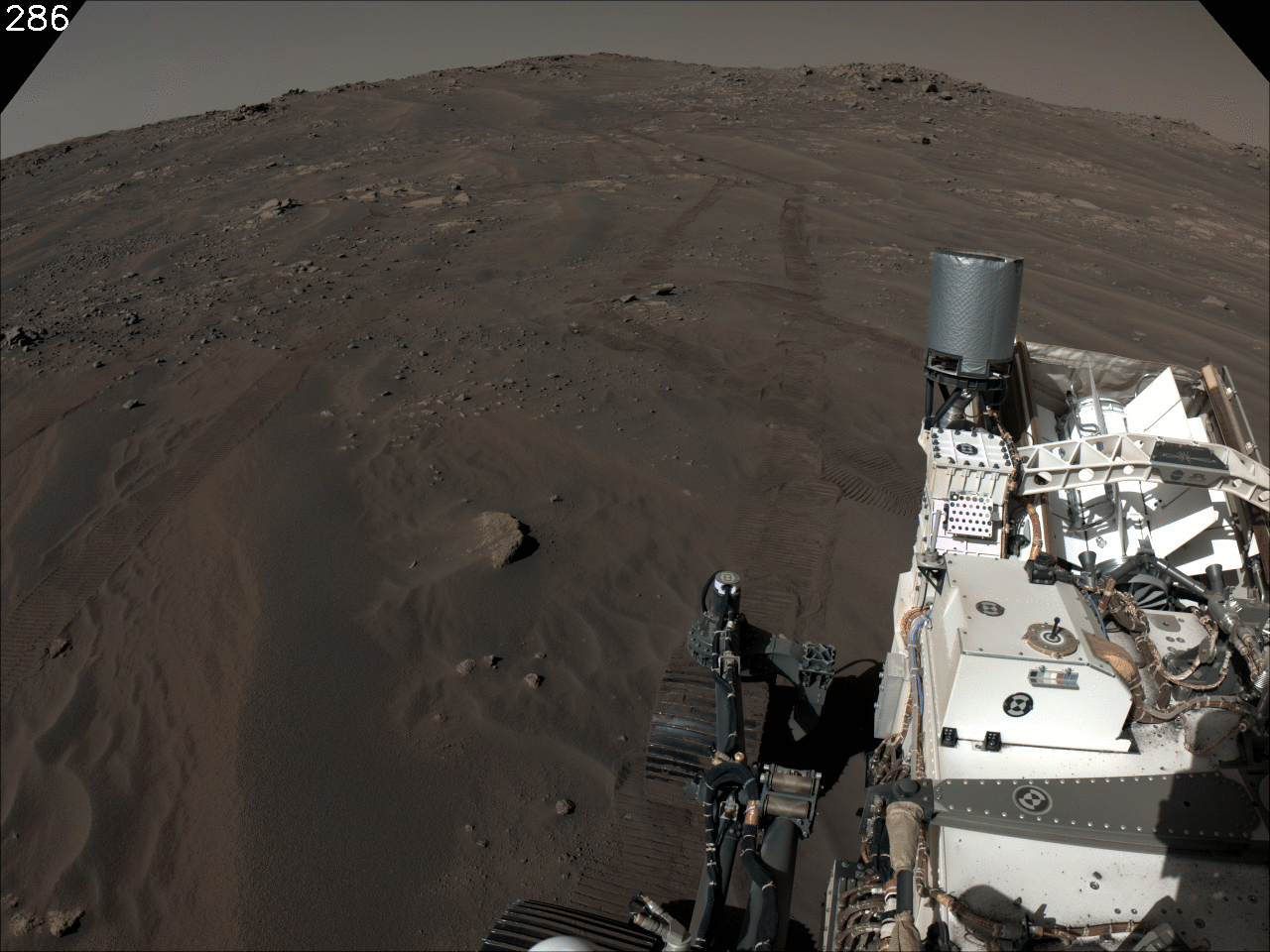

Supplement: Supplementary file 9 — Movie S8 [file GRL-49-e2022GL100126-s002.gif]

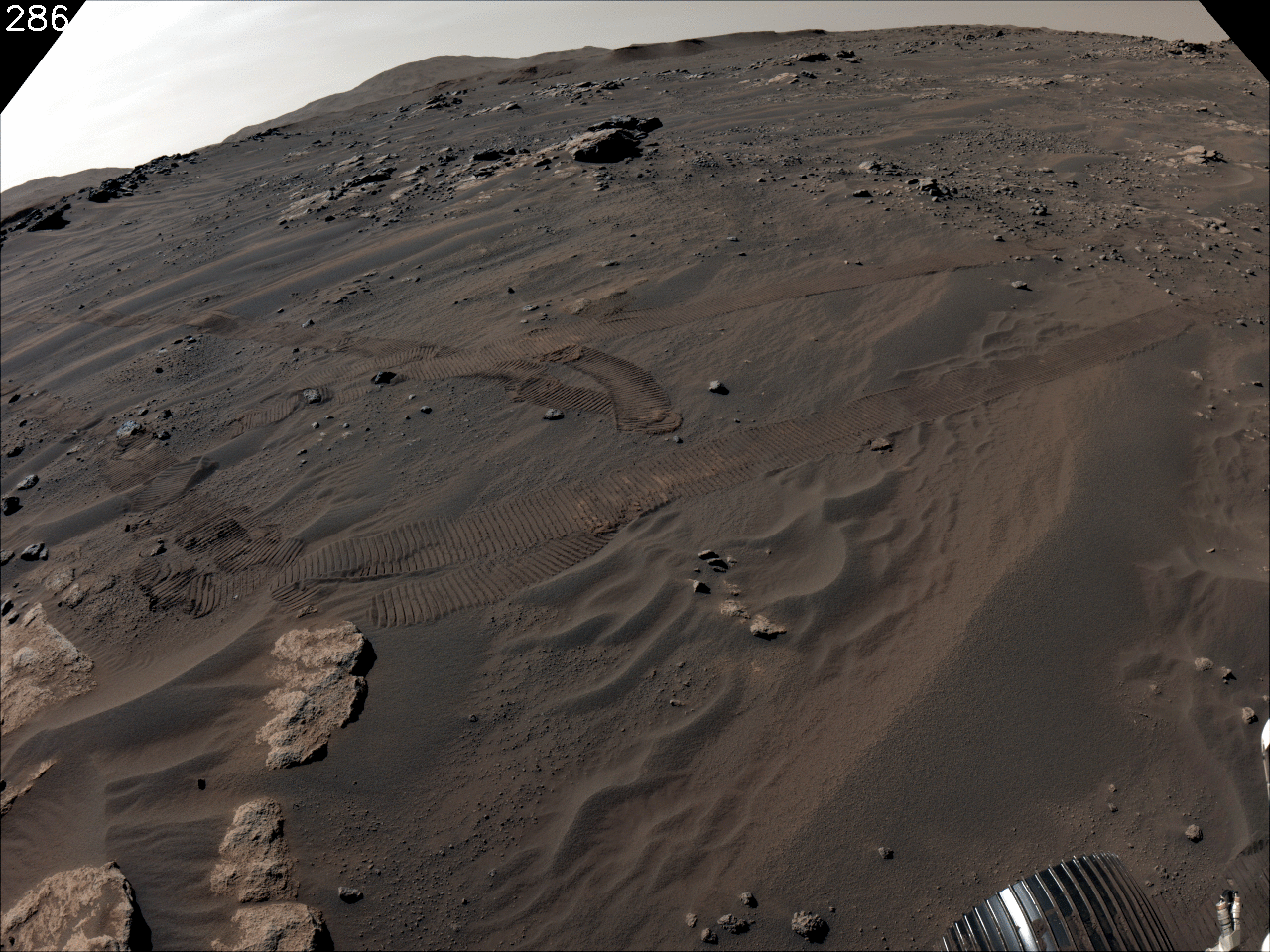

Supplement: Supplementary file 10 — Movie S9 [file GRL-49-e2022GL100126-s006.gif]

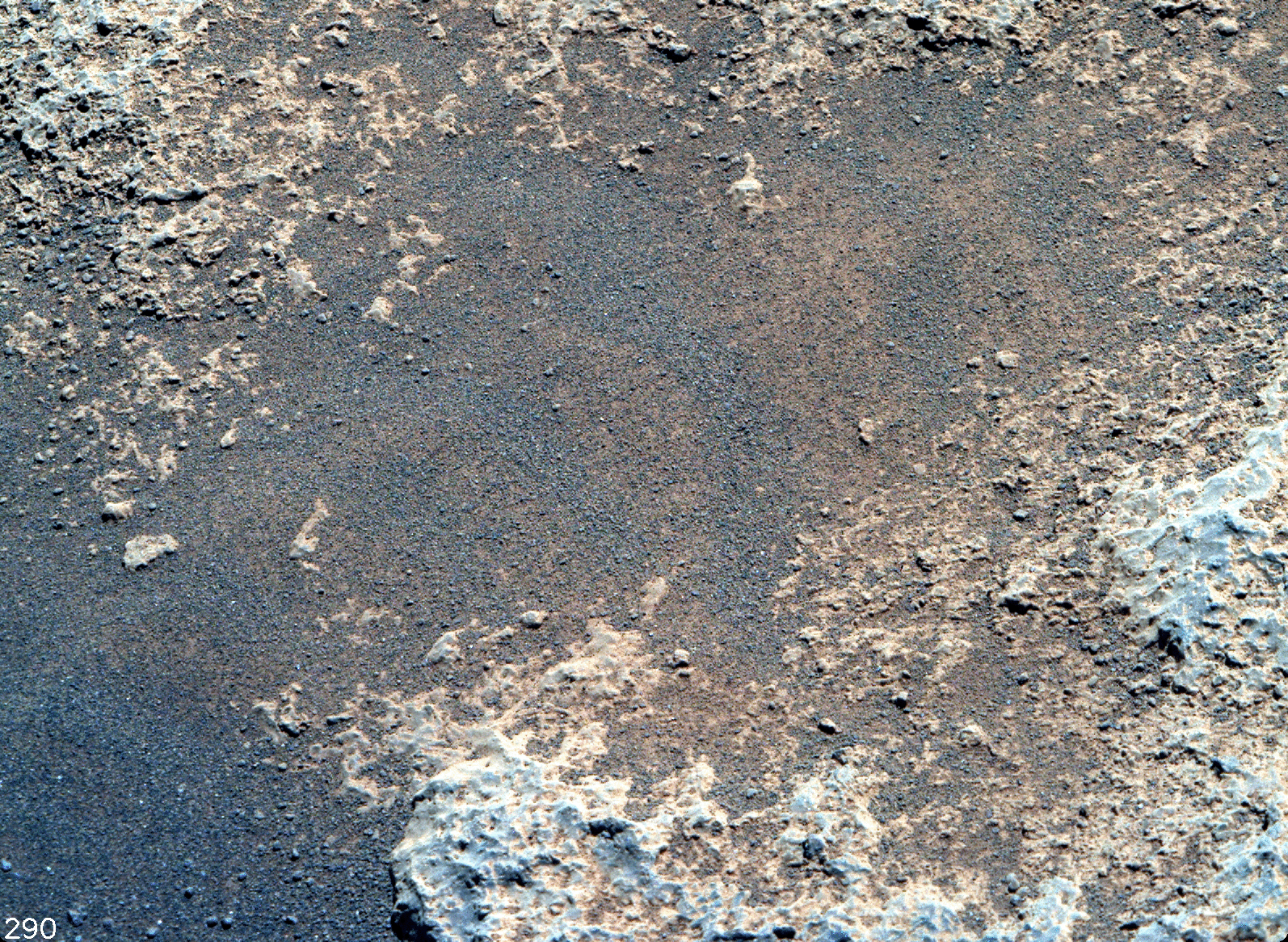

Supplement: Supplementary file 11 — Movie S10 [file GRL-49-e2022GL100126-s003.gif]

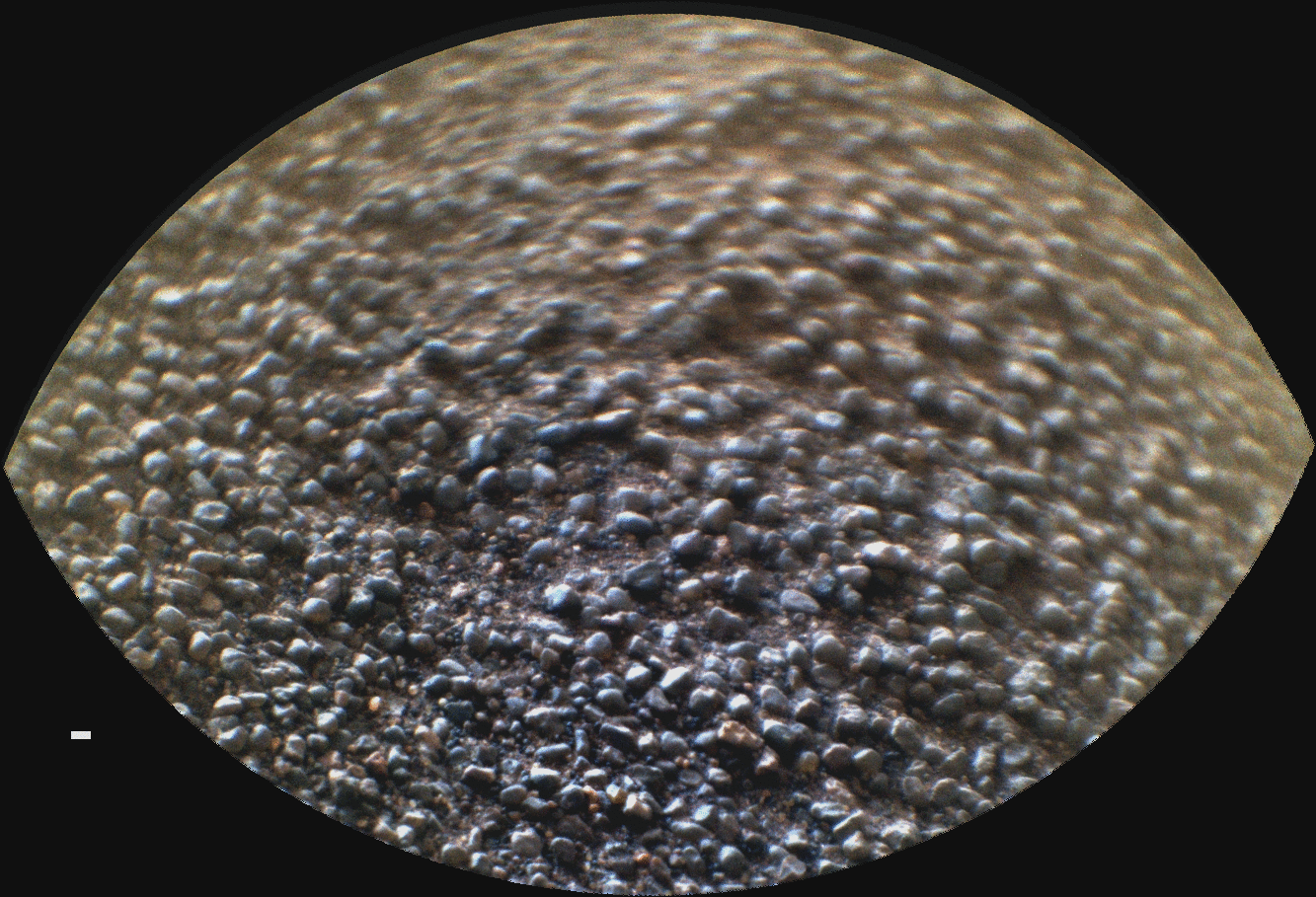

Supplement: Supplementary file 12 — Movie S11 [file GRL-49-e2022GL100126-s005.gif]
